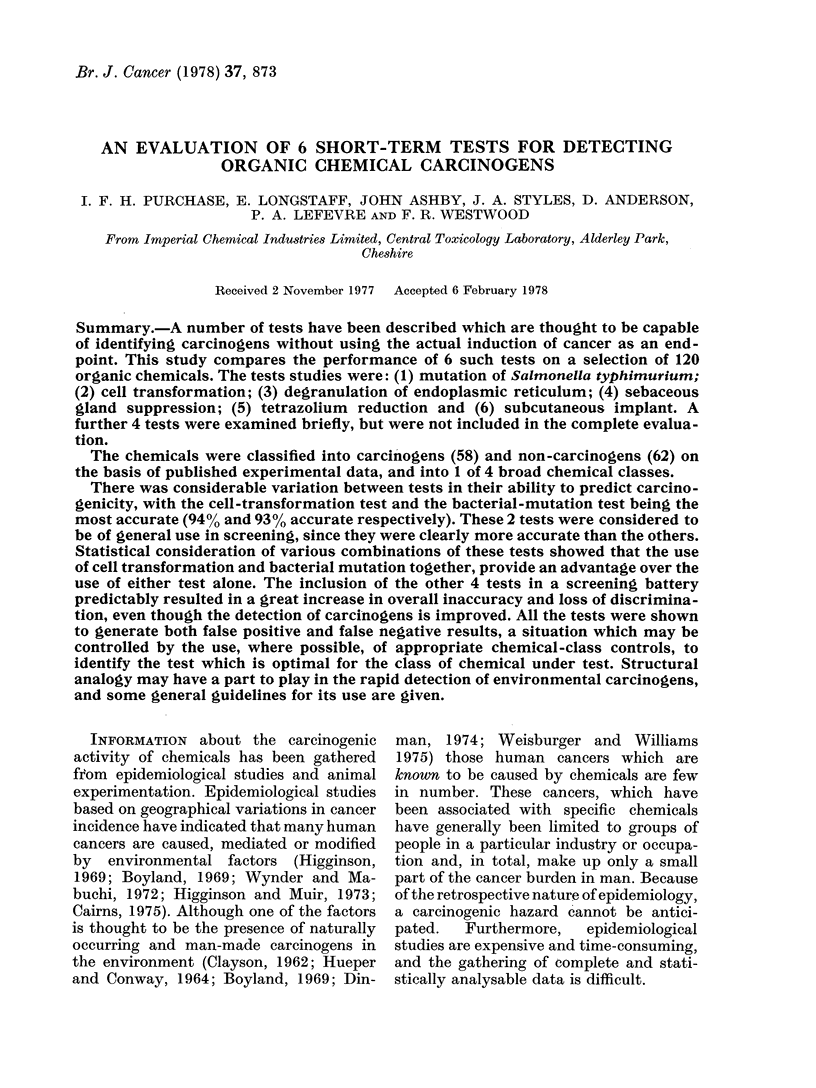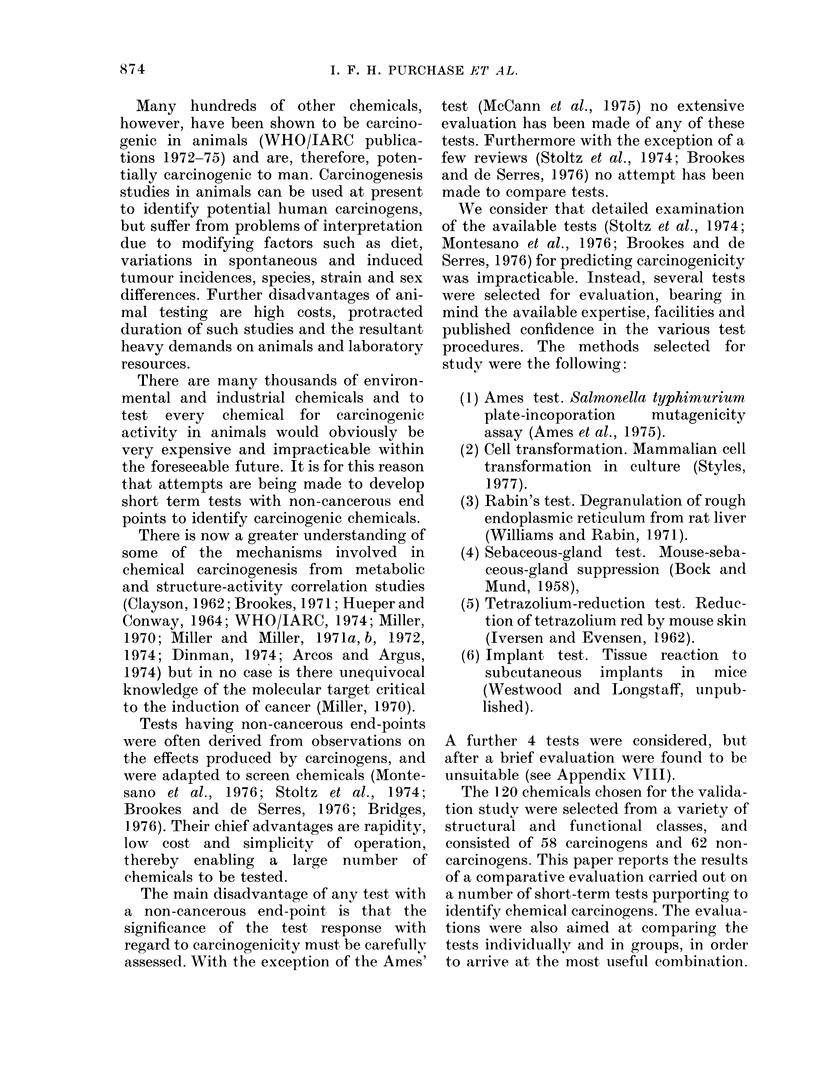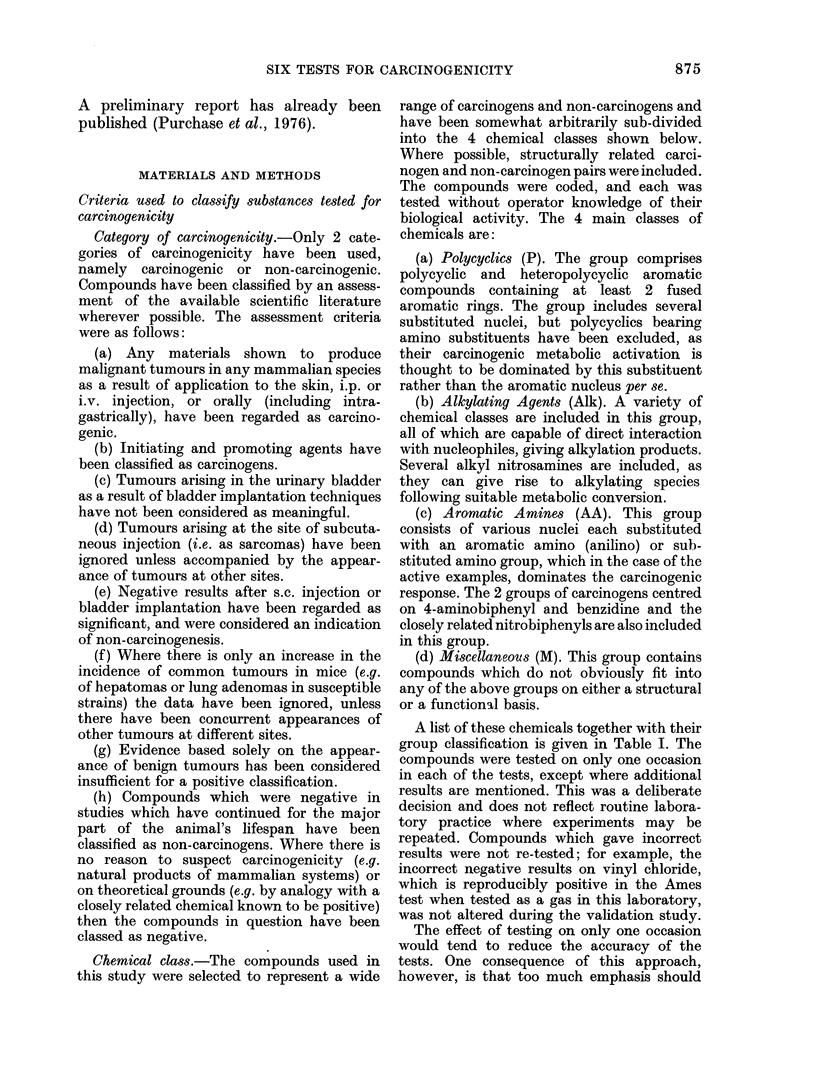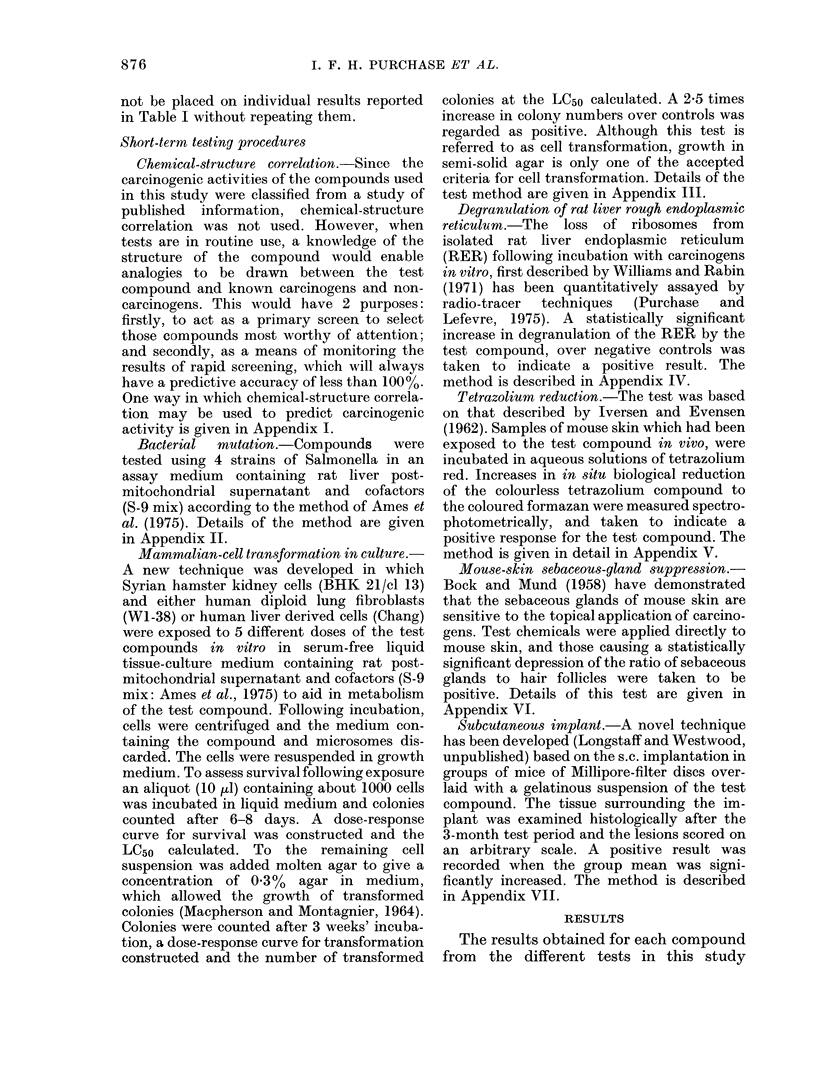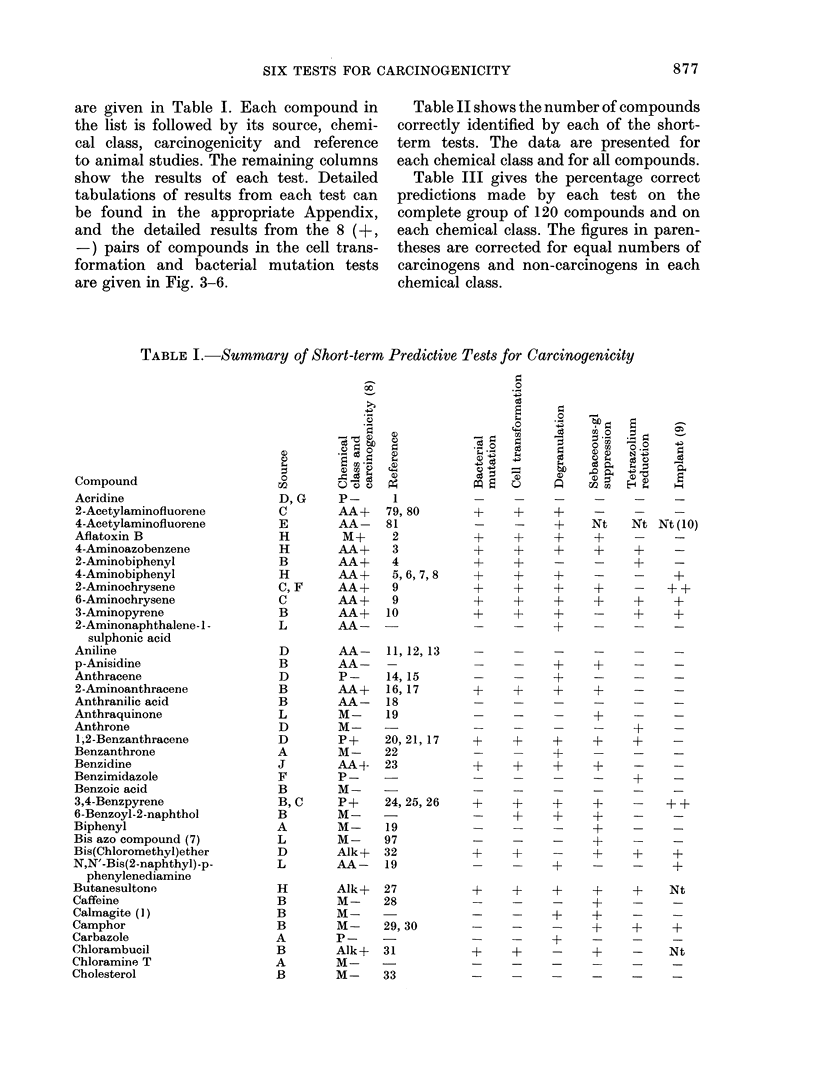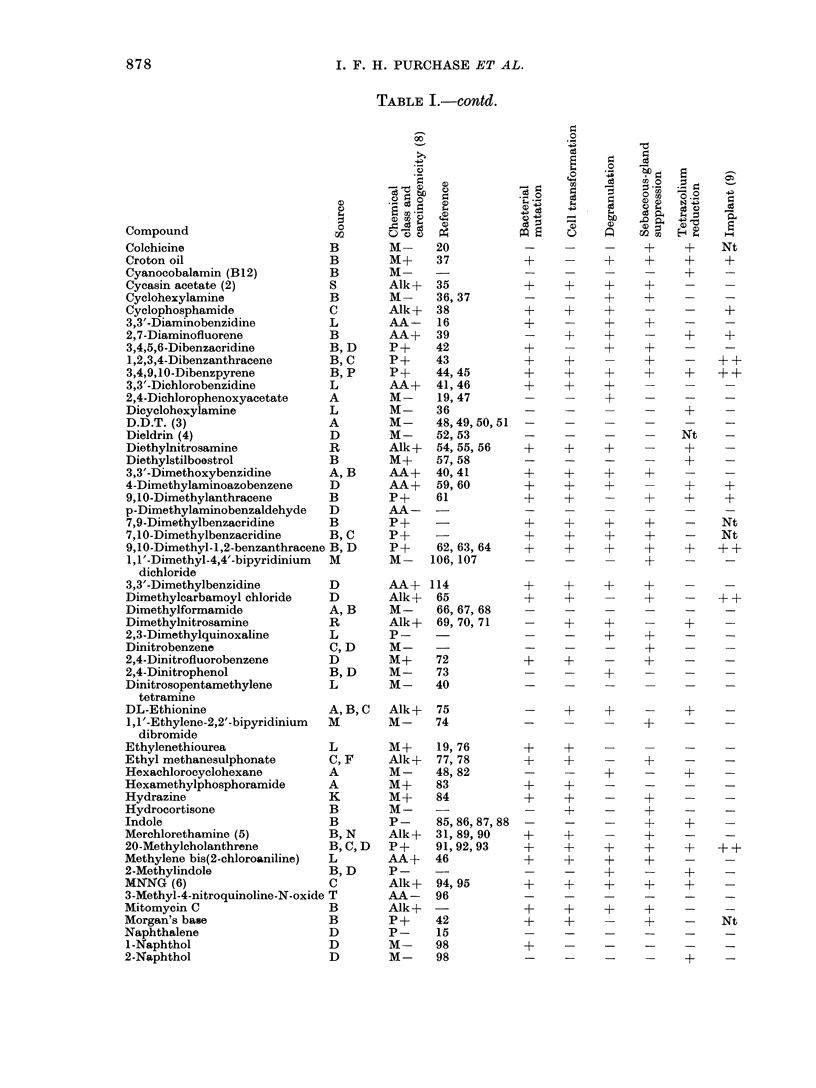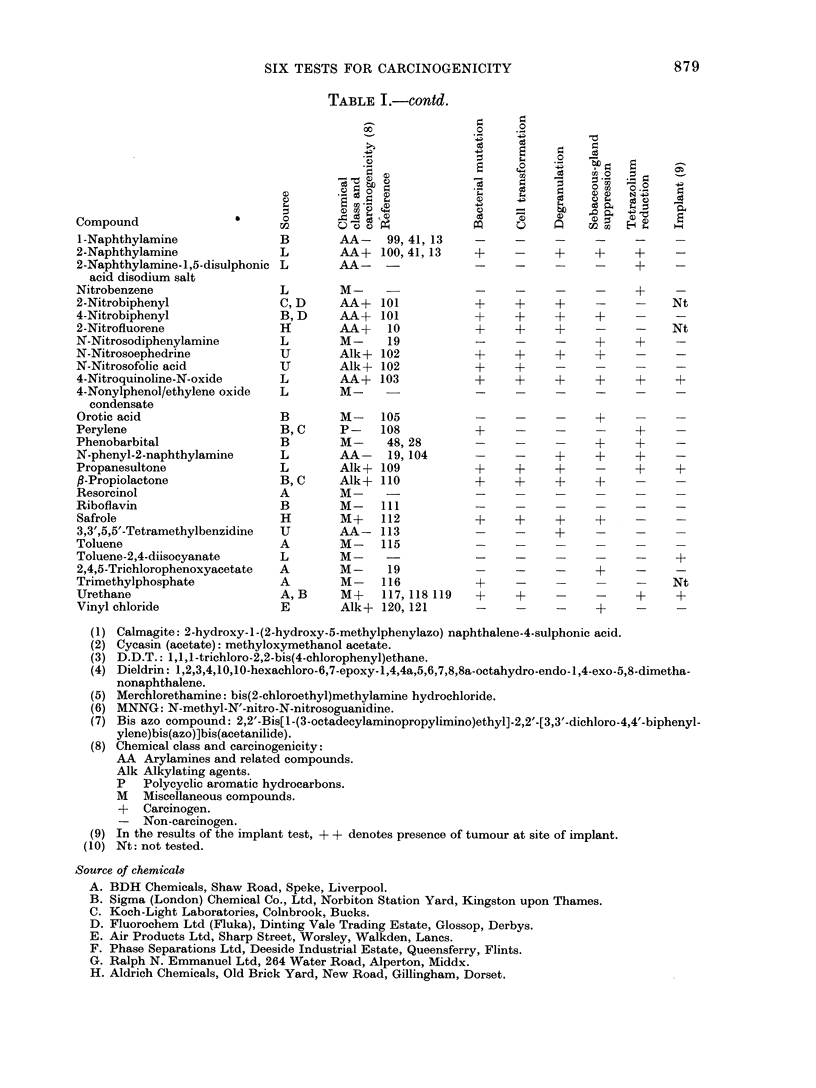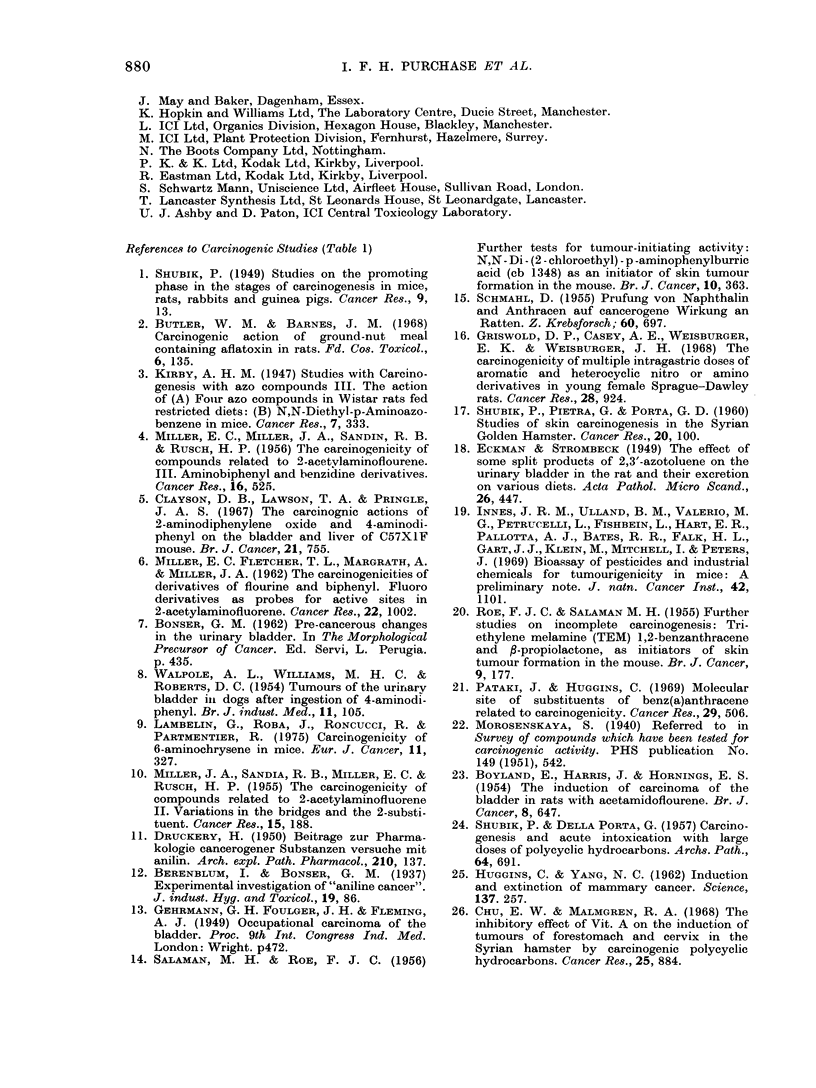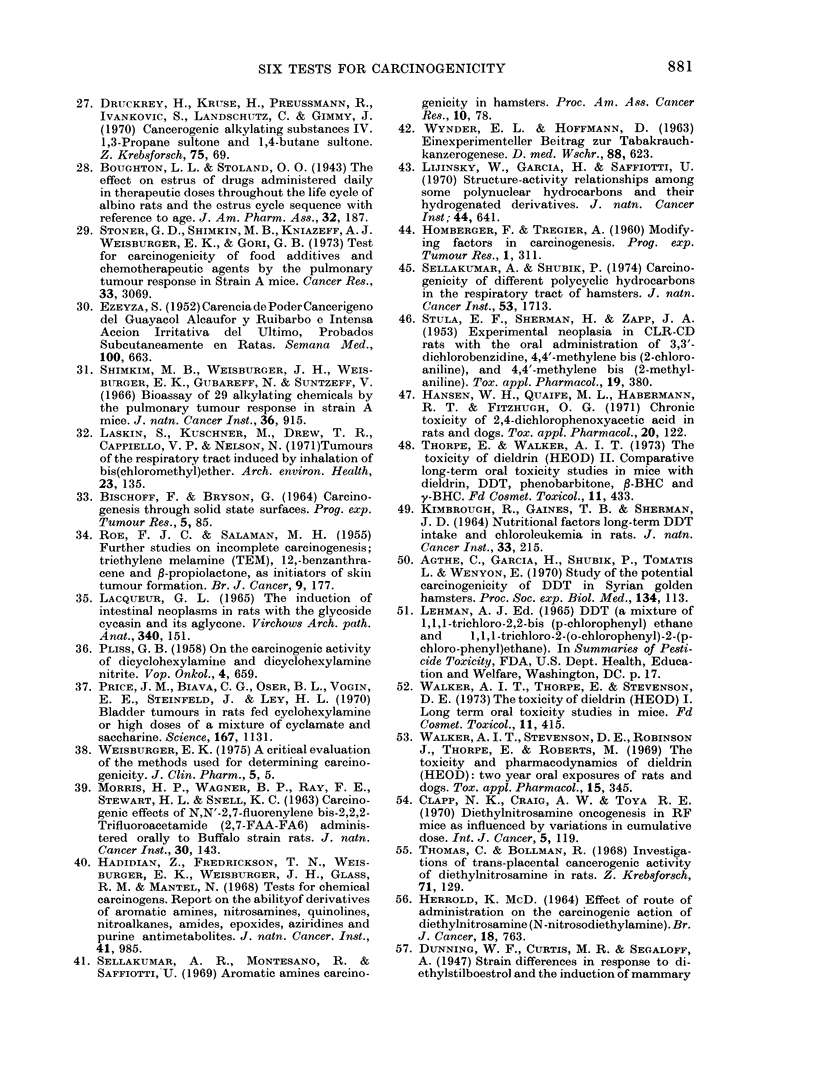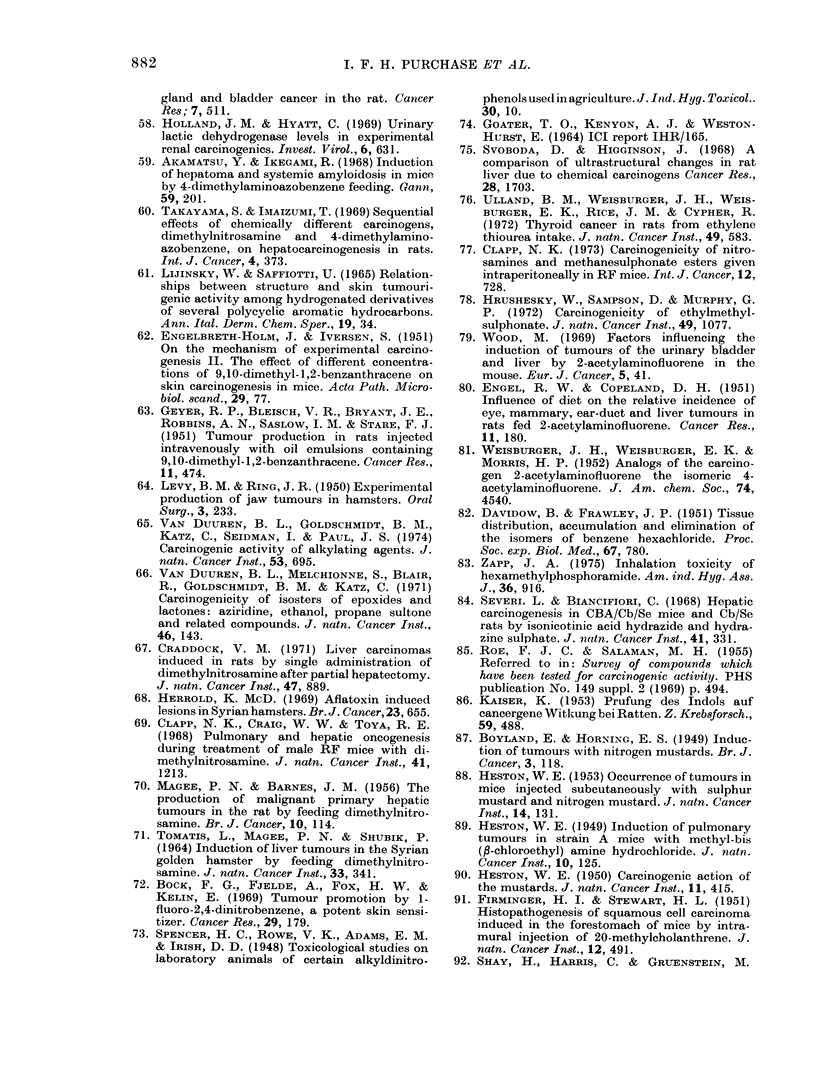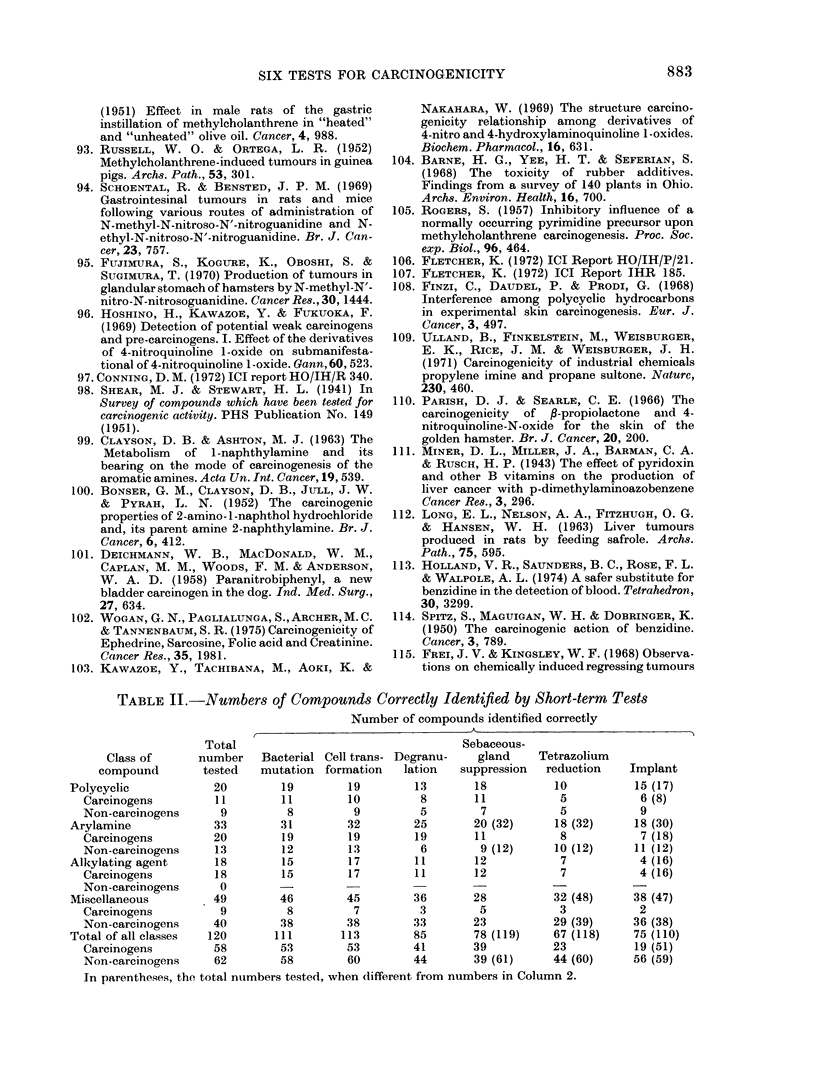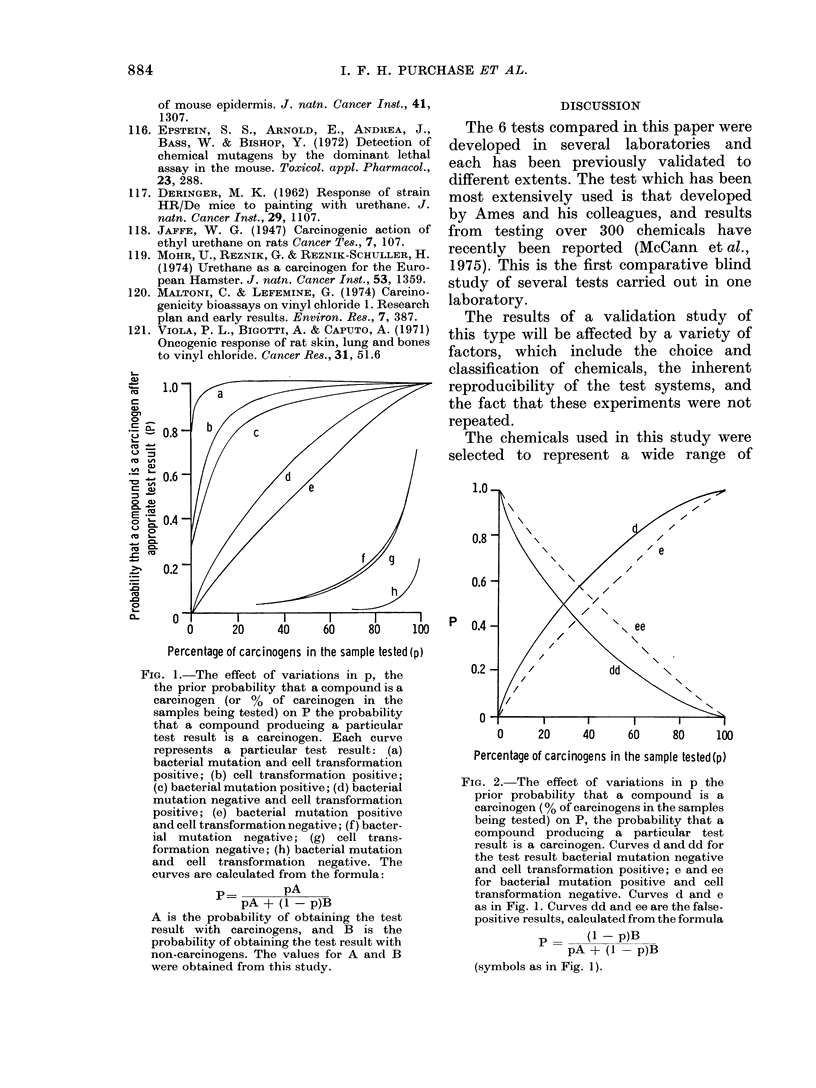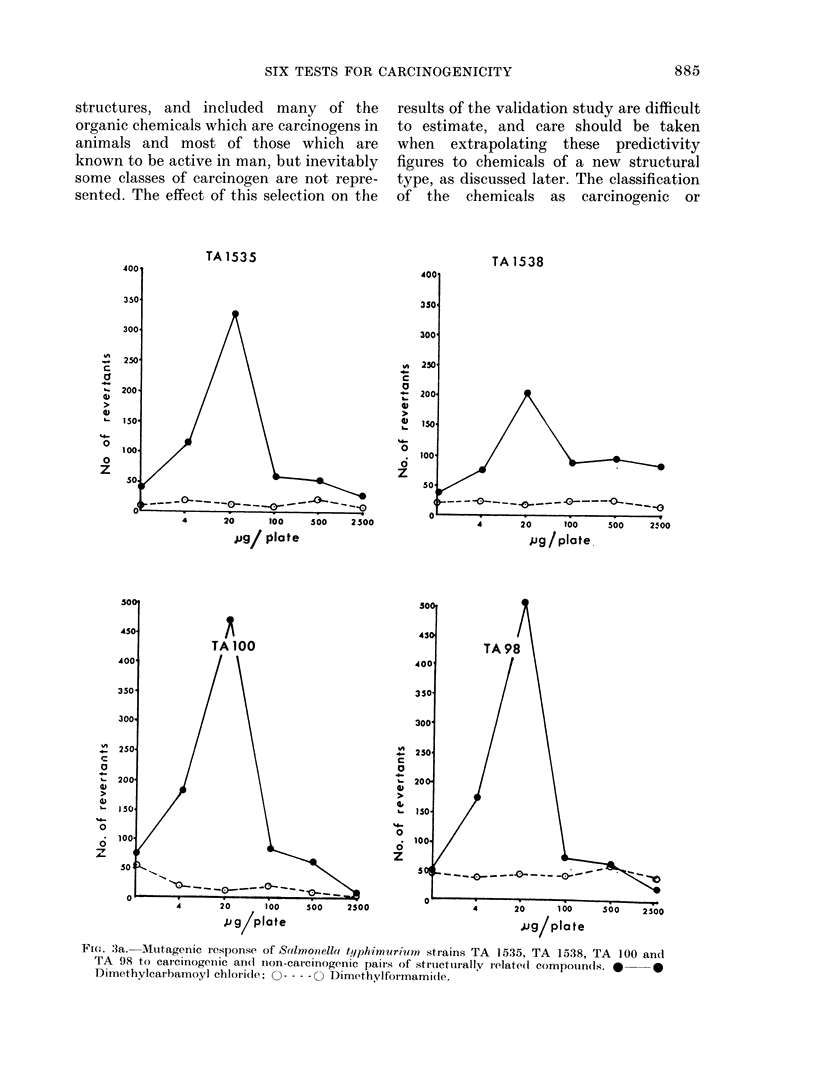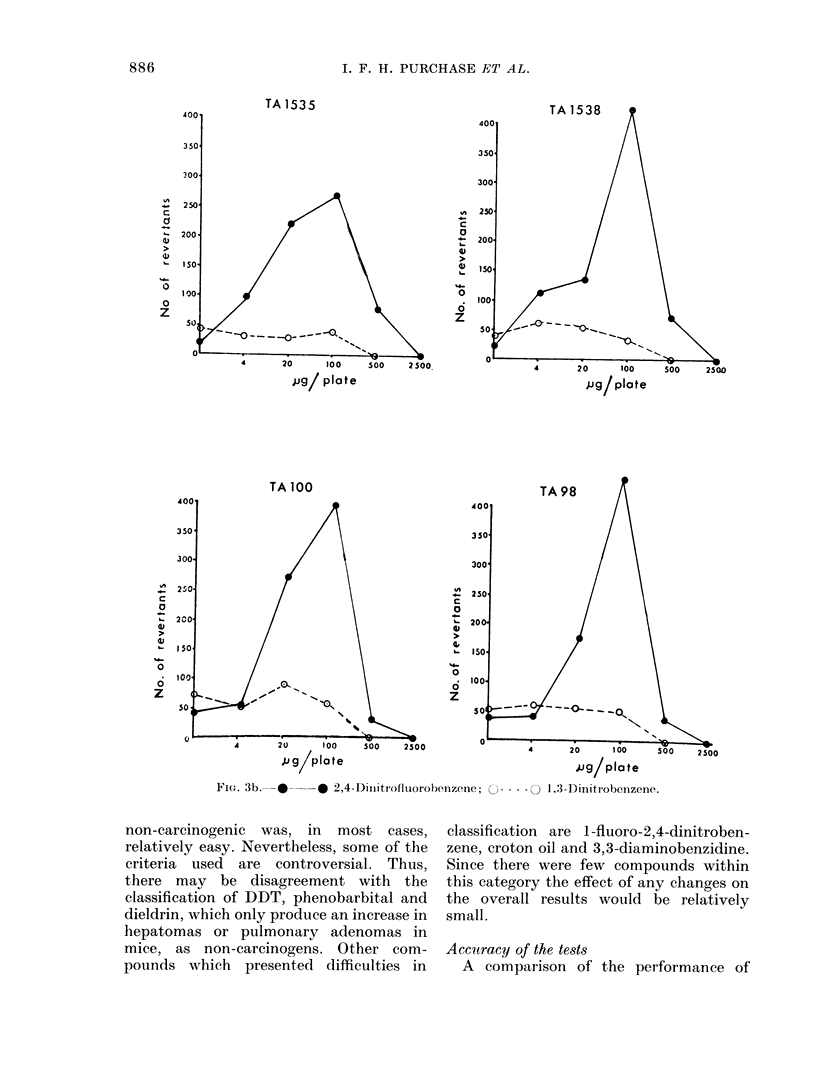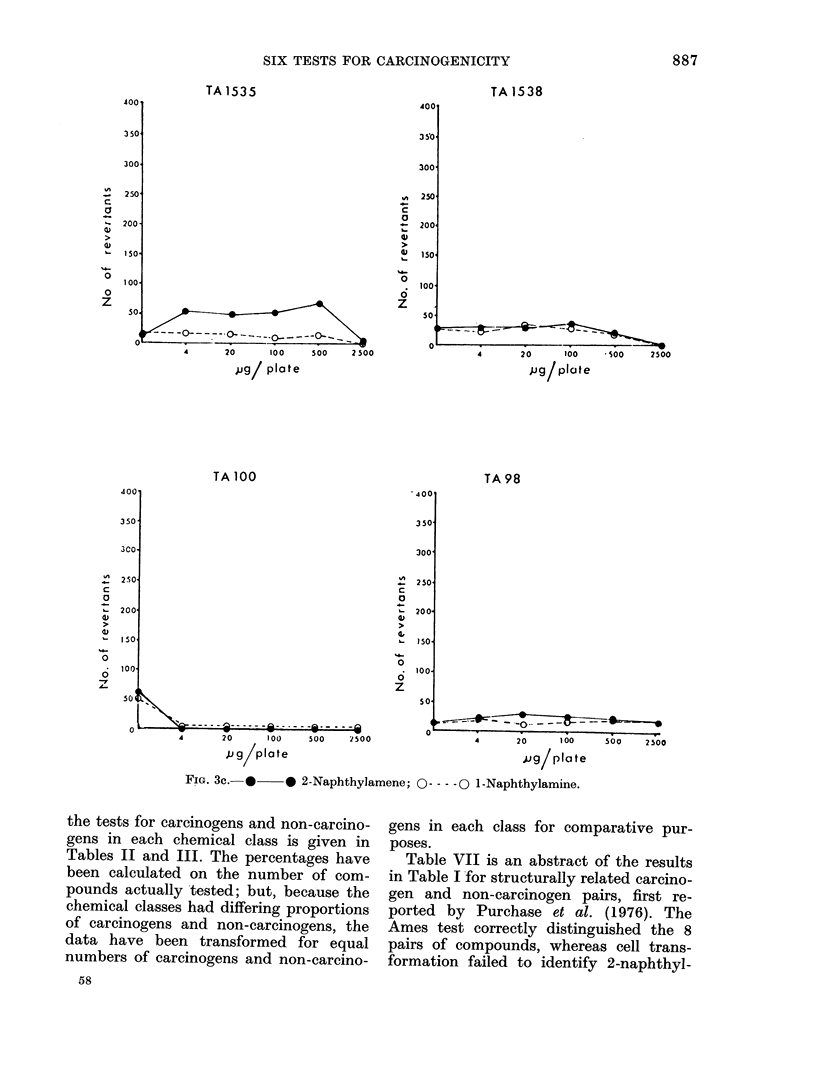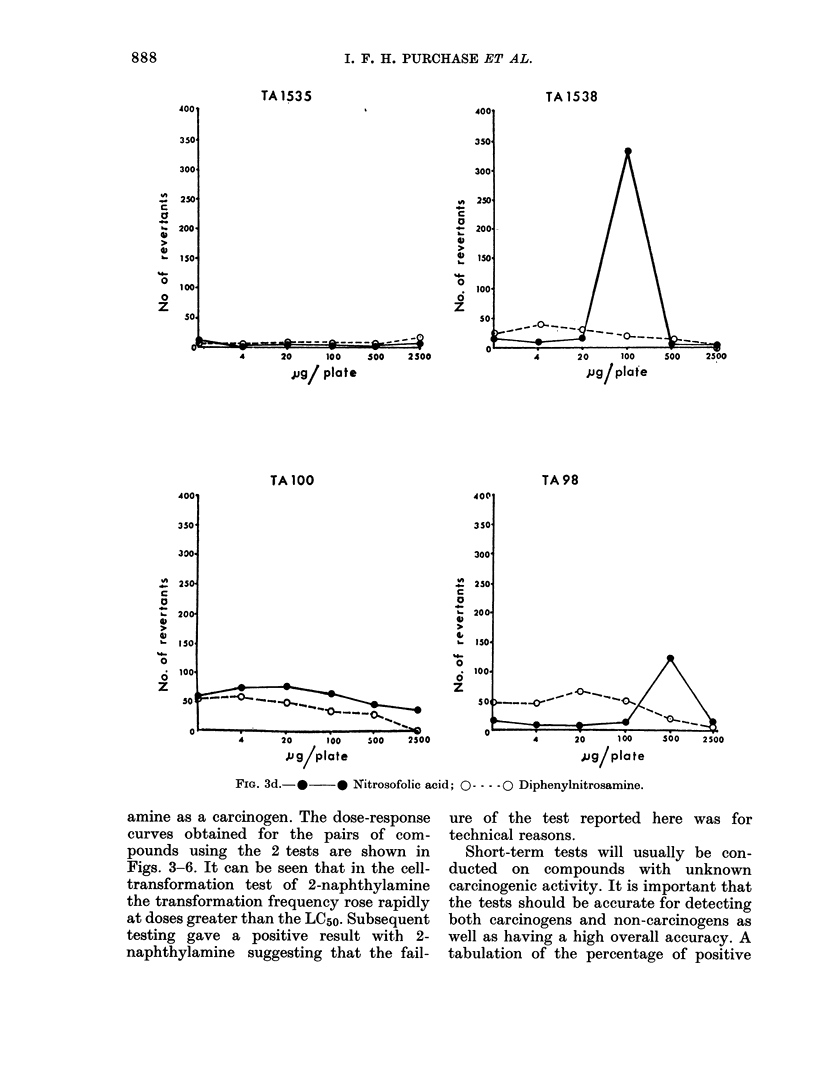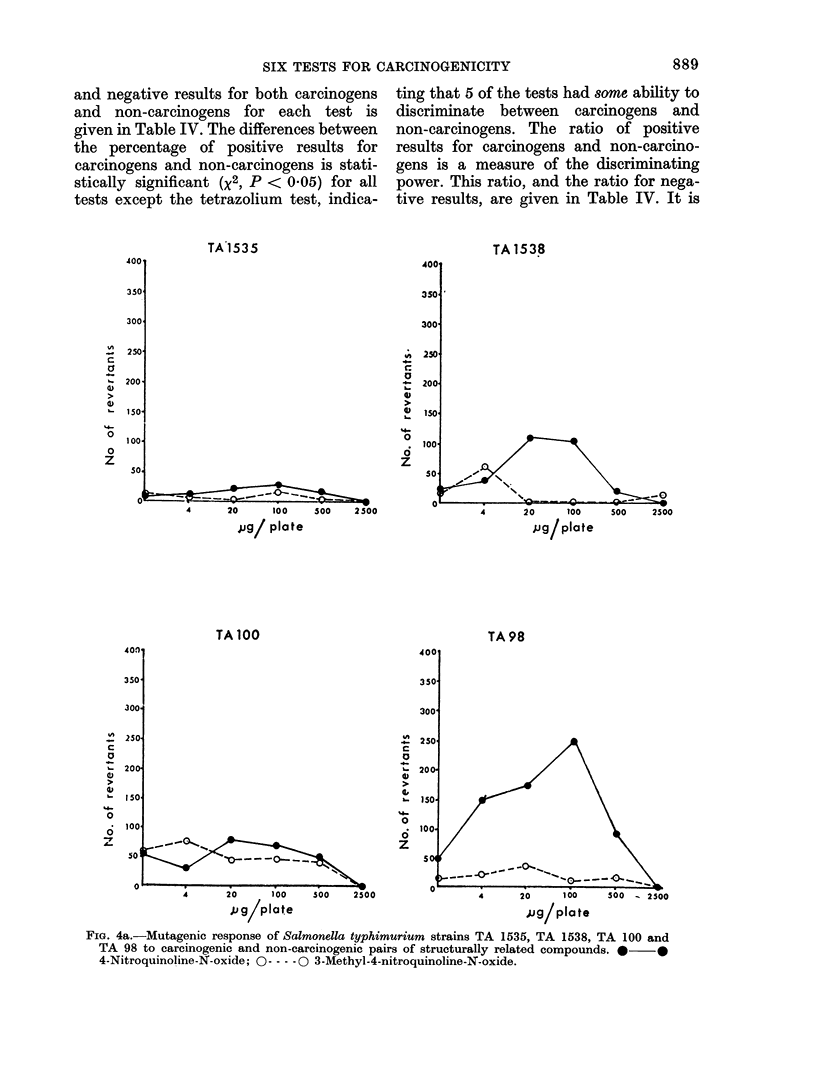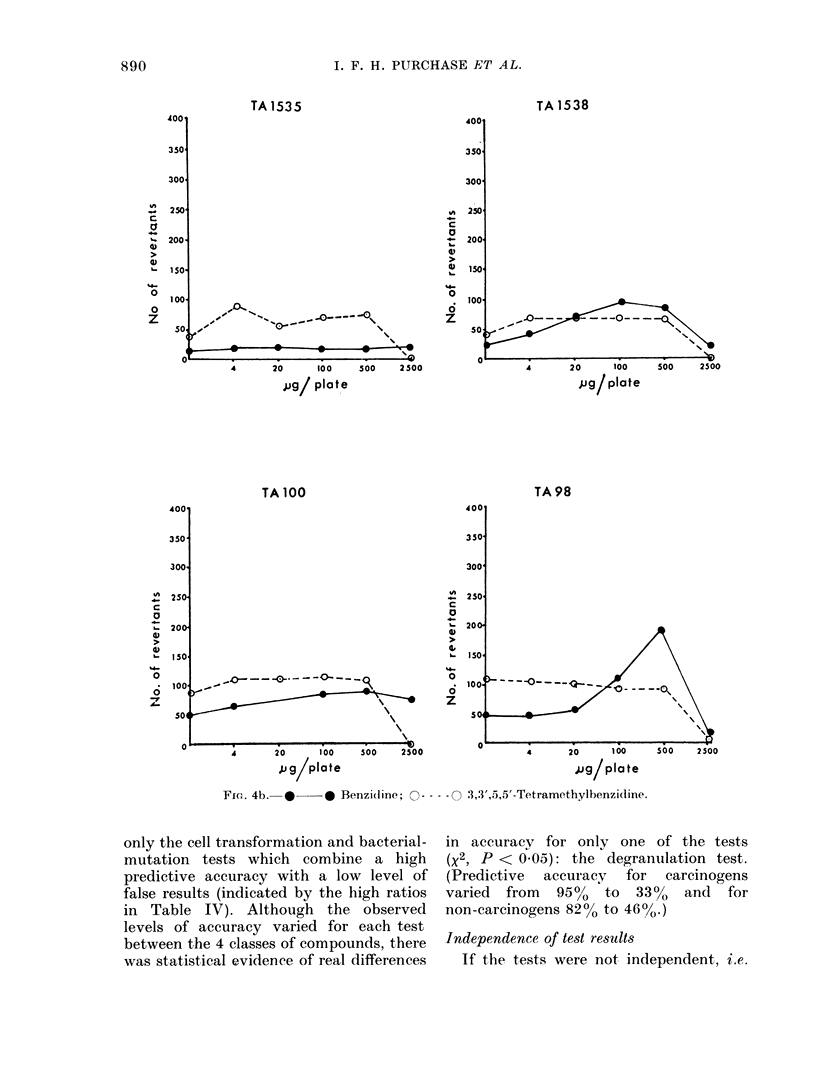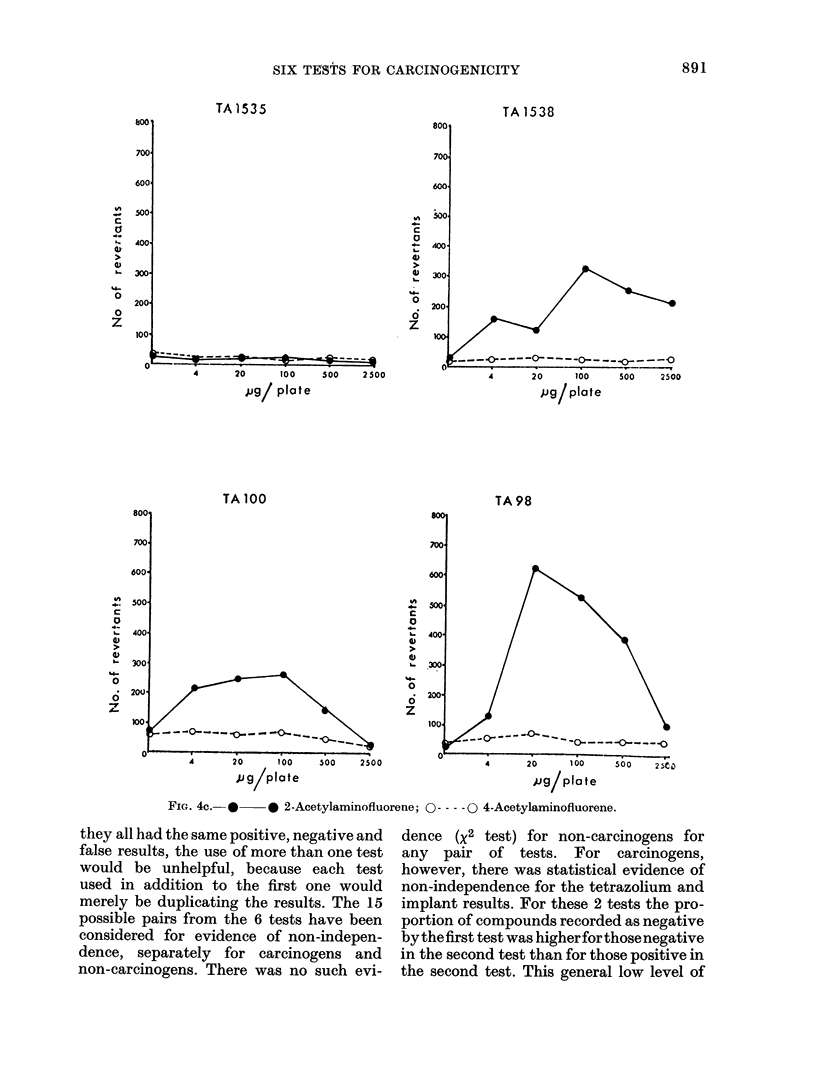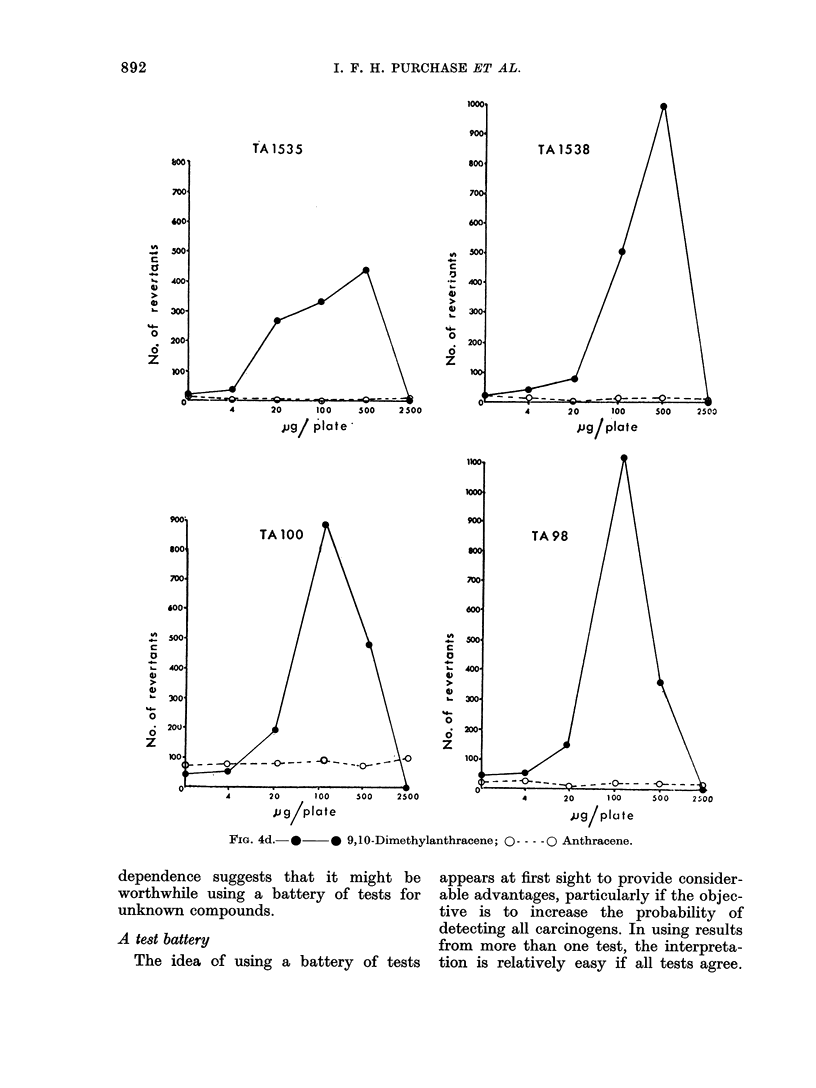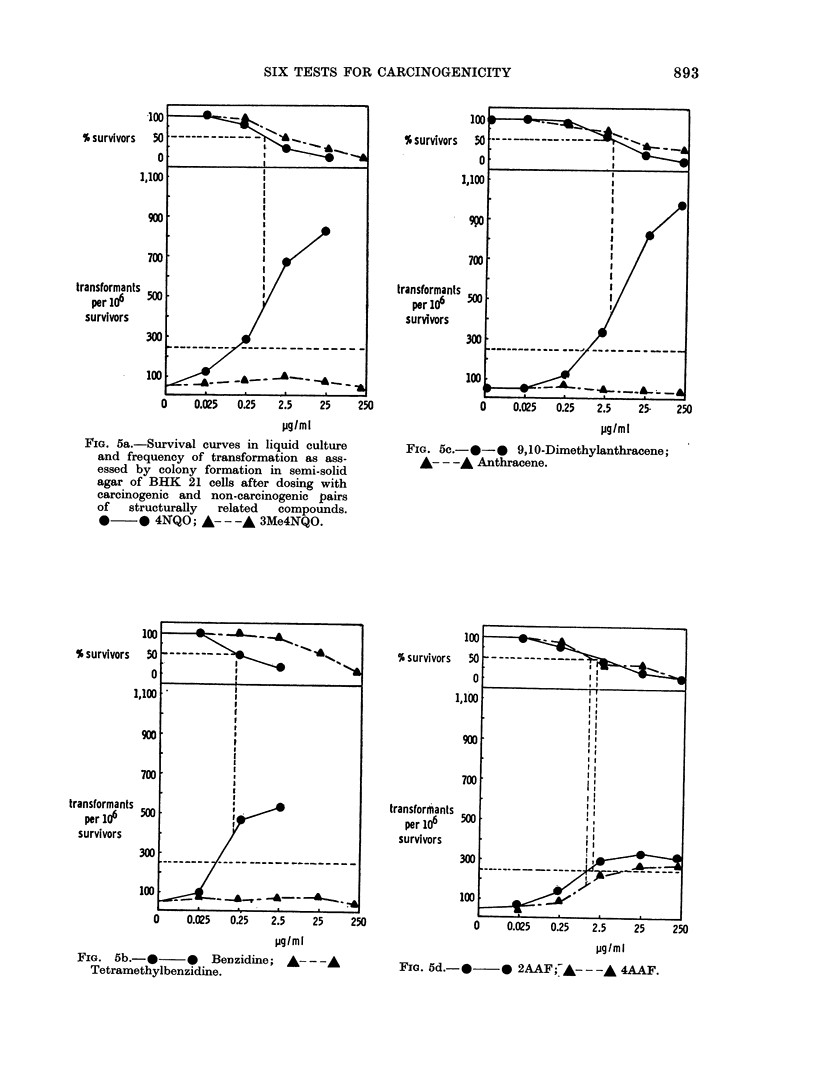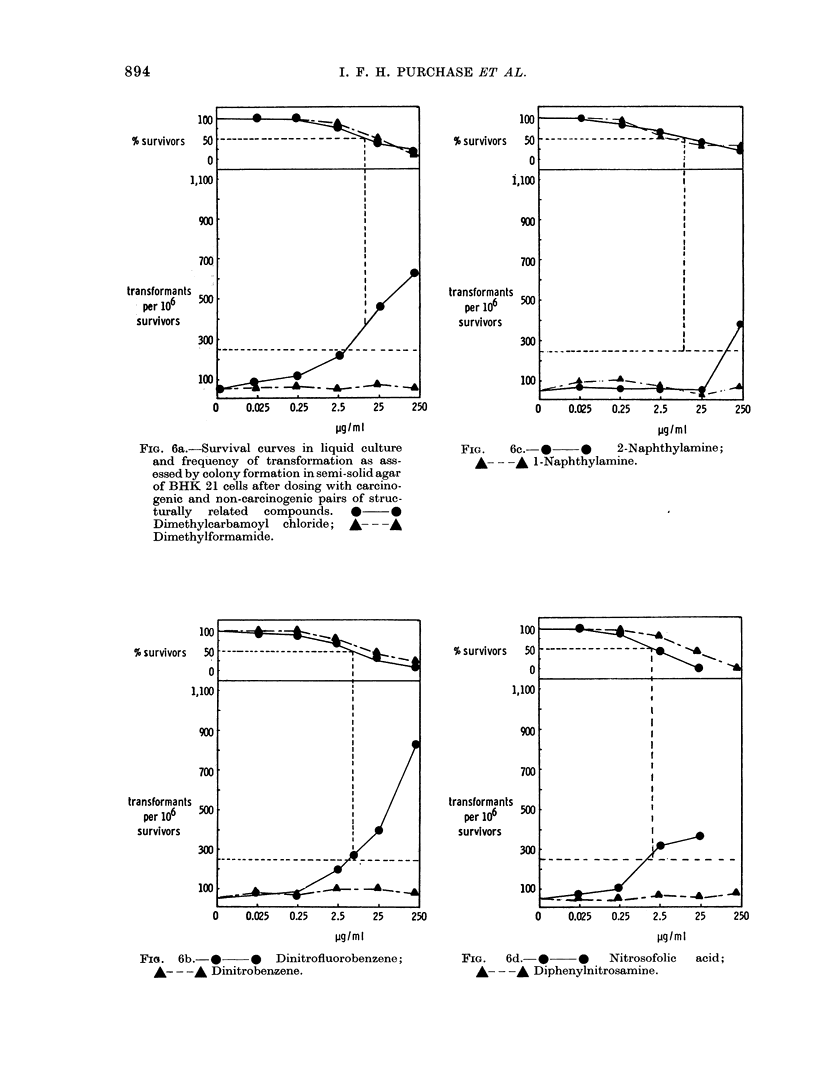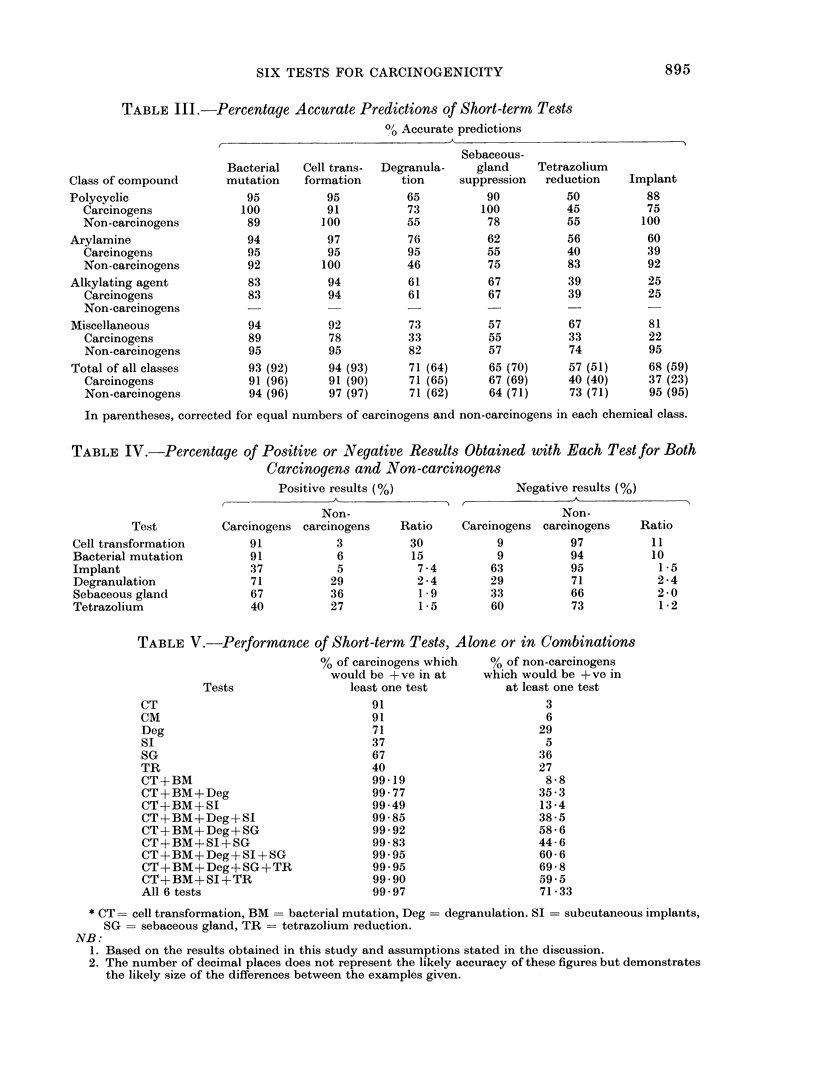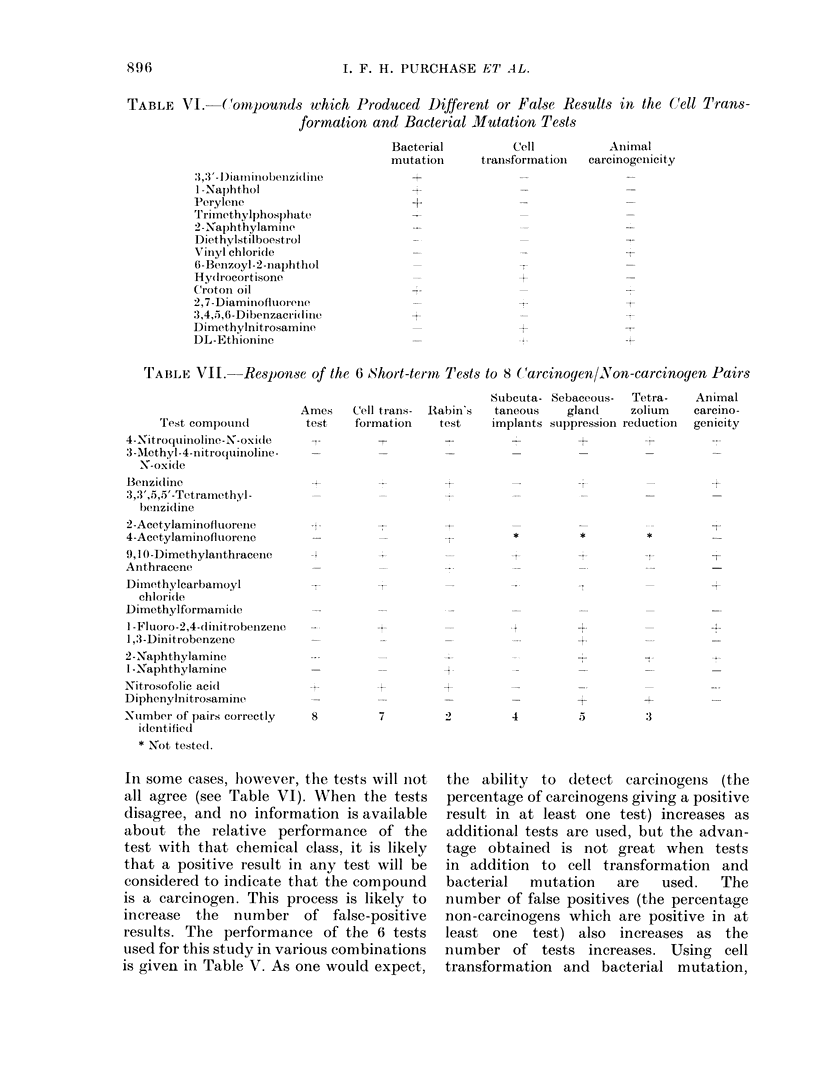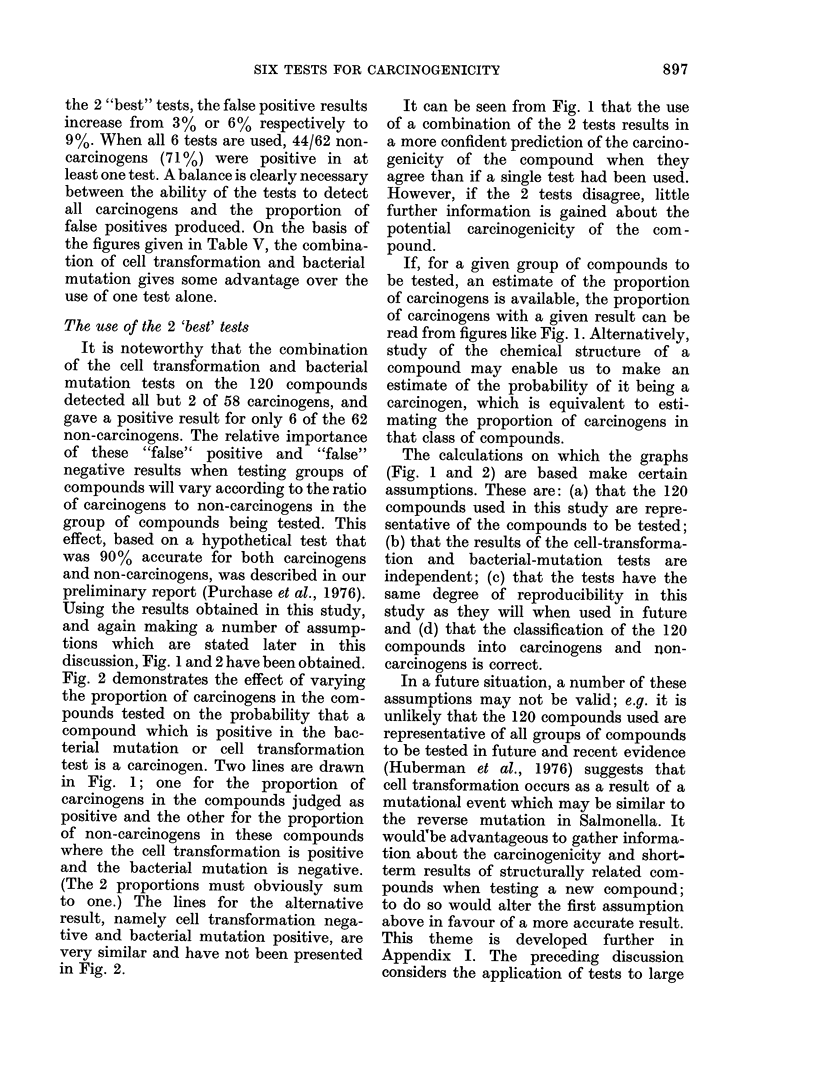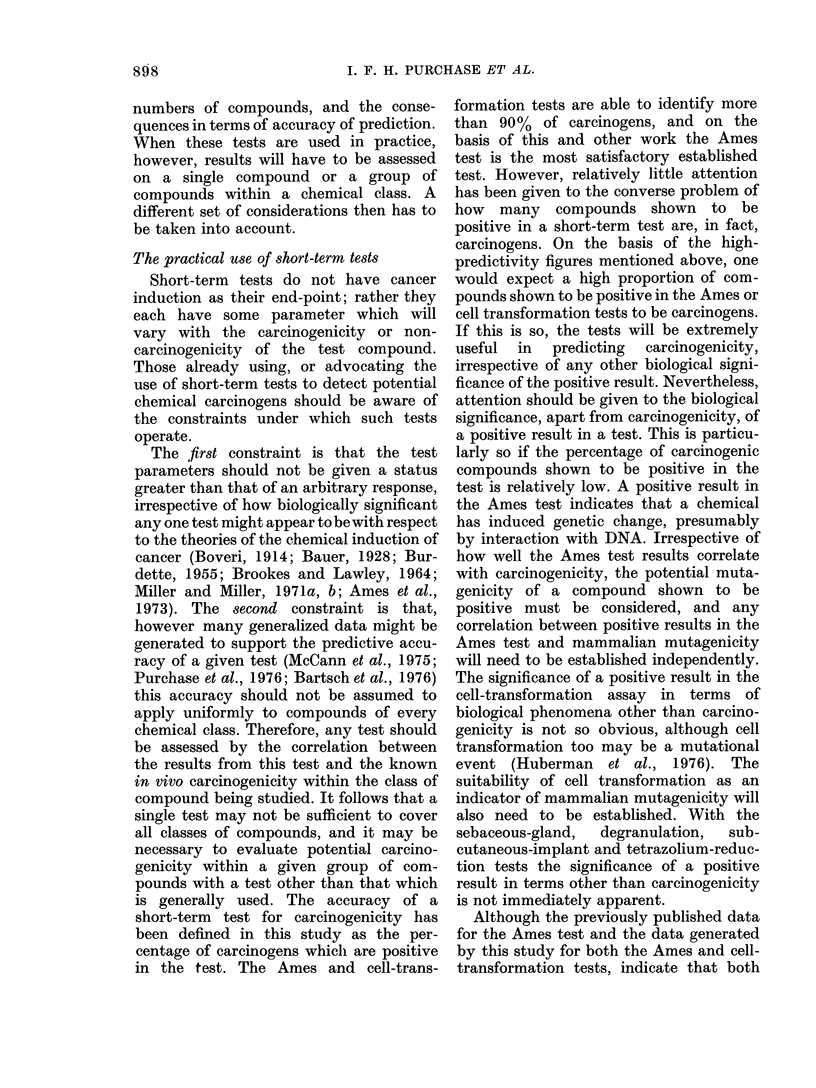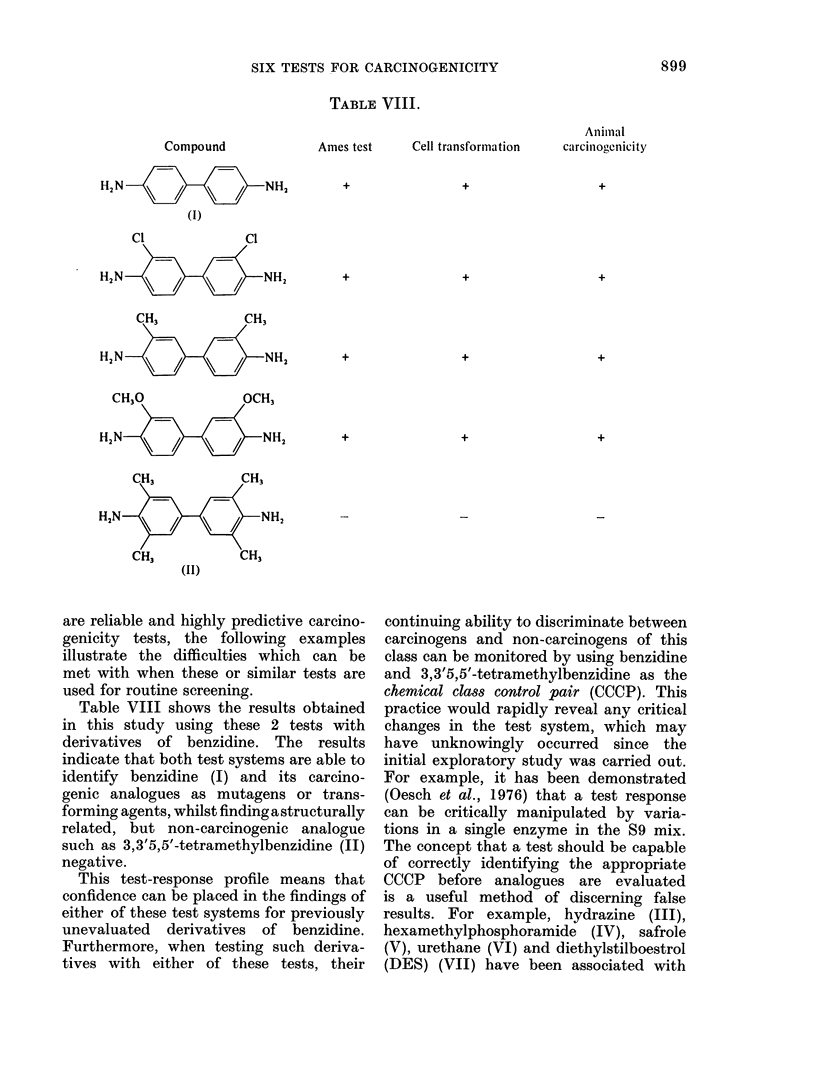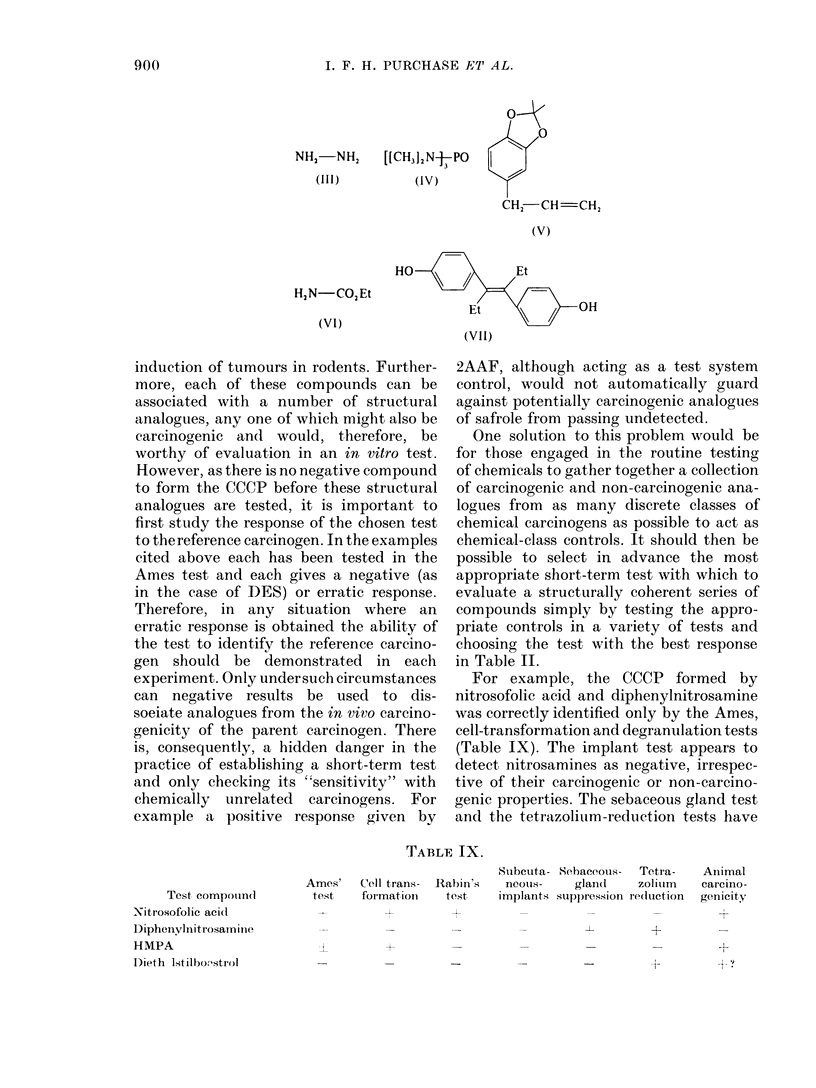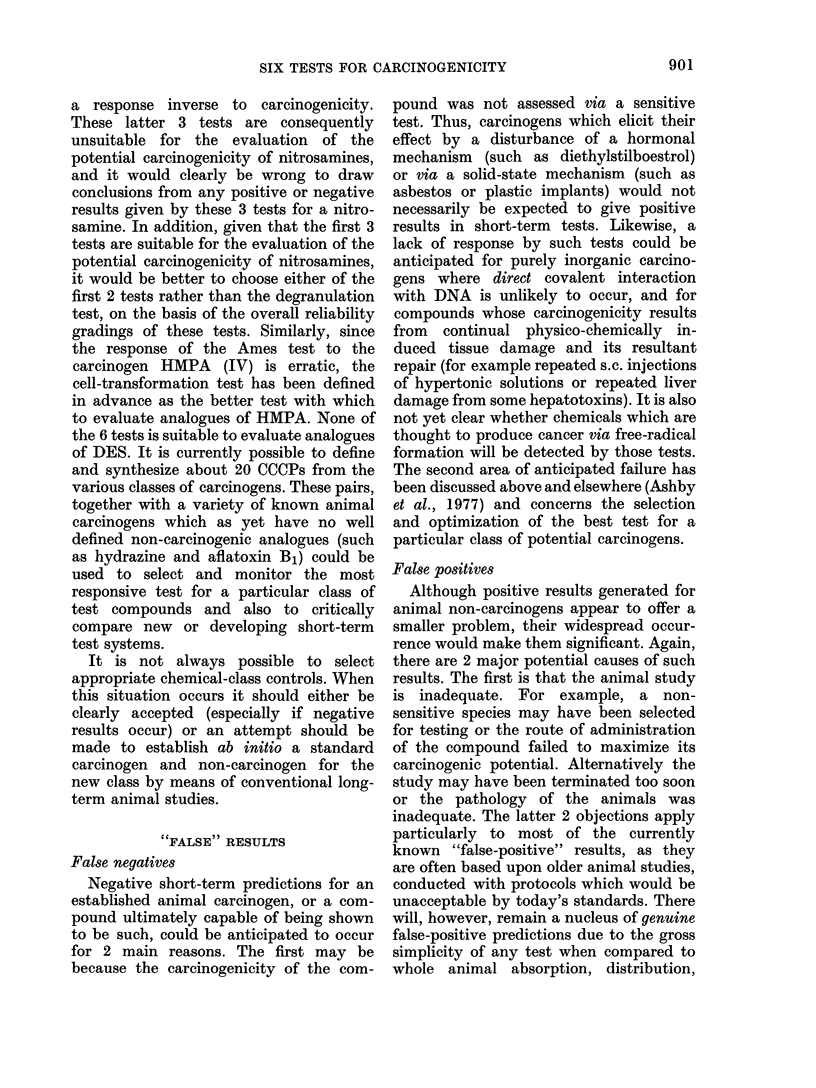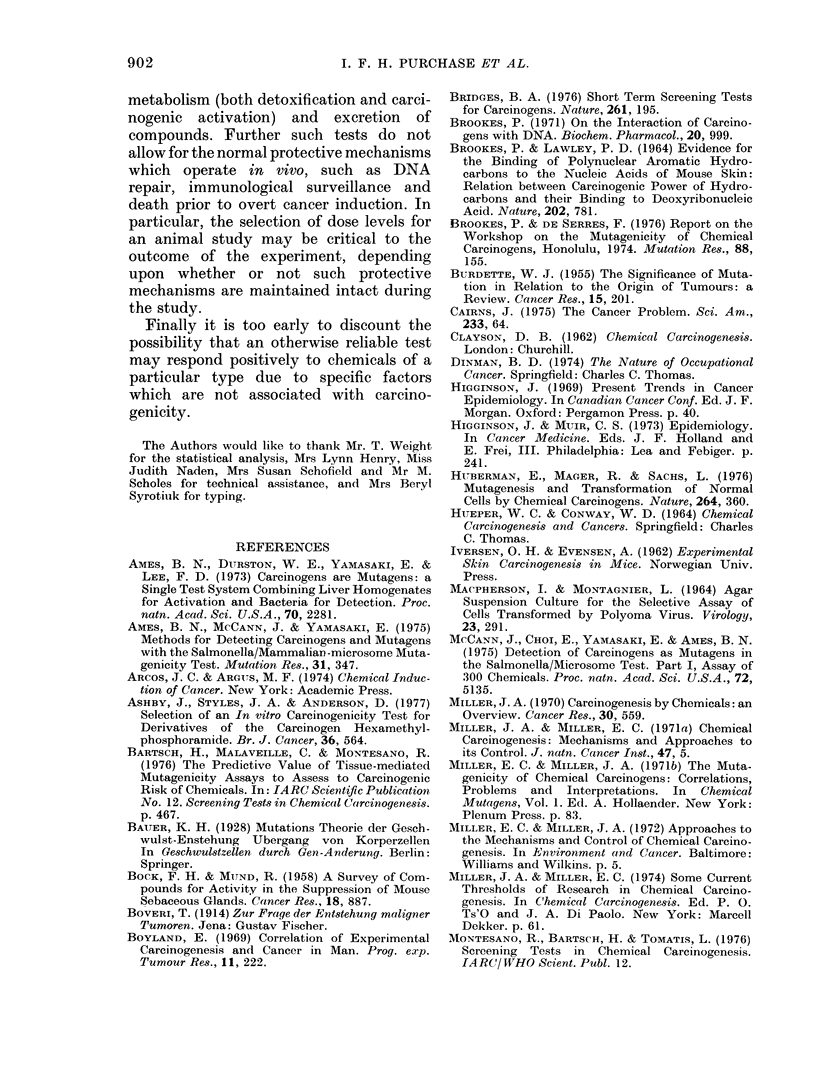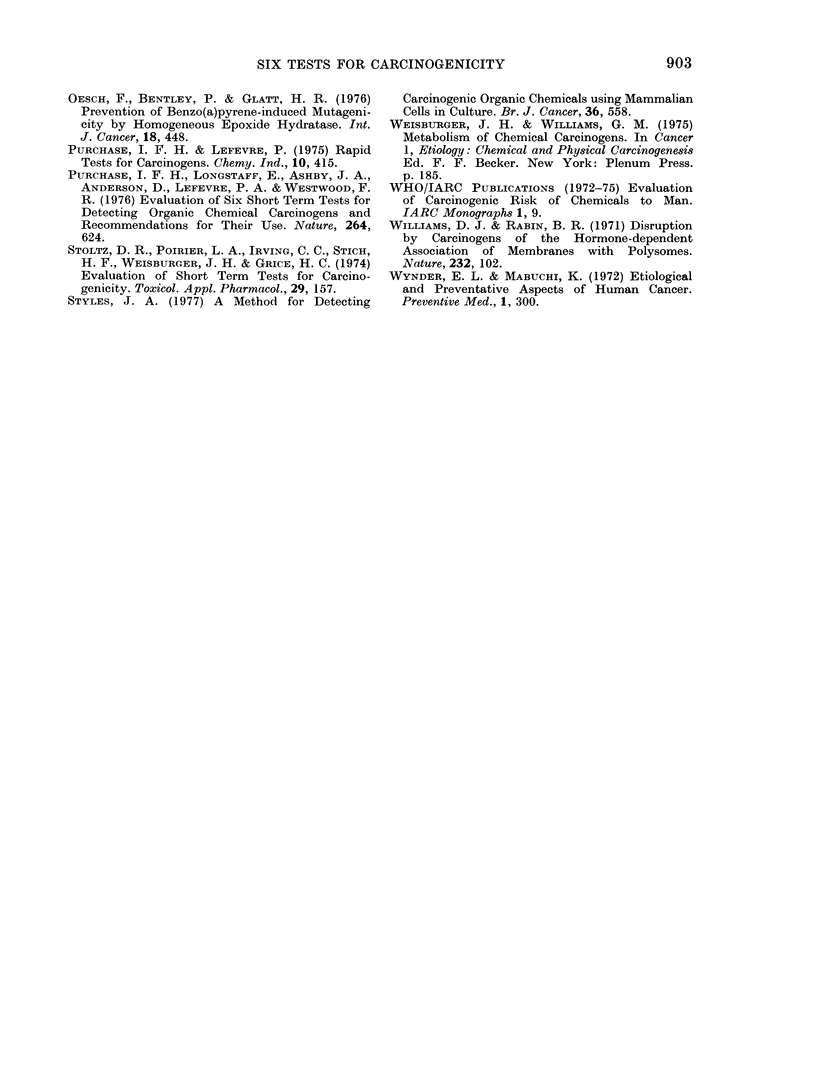# An evaluation of 6 short-term tests for detecting organic chemical carcinogens.

**DOI:** 10.1038/bjc.1978.132

**Published:** 1978-06

**Authors:** I. F. Purchase, E. Longstaff, J. Ashby, J. A. Styles, D. Anderson, P. A. Lefevre, F. R. Westwood

## Abstract

A number of tests have been described which are thought to be capable of identifying carcinogens without using the actual induction of cancer as an endpoint. This study compared the performance of 6 such tests on a selection of 120 organic chemicals. The tests studies were: (1) mutation of Salmonella typhimurium; (2) cell transformation; (3) degranulation of endoplasmic reticulum; (4) sebaceous gland suppression; (5) tetrazolium reduction and (6) subcutaneous implant. A further 4 tests were examined briefly, but were not included in the complete evaluation. The chemicals were classified into carcinogens (58) and non-carcinogens (62) on the basis of published experimental data, and into 1 of 4 broad chemical classes. There was considerable variation between tests in their ability to predict carcinogenicity, with the cell-transformation test and the bacterial-mutation test being the most accurate (94% and 93% accurate respectively). These 2 tests were considered to be of general use in screening, since they were clearly more accurate than the others. Statistical consideration of various combinations of these tests showed that the use of cell transformation and bacterial mutation together, provide an advantage over the use of either test alone. The inclusion of the other 4 tests in a screening battery predictably resulted in a great increase in overall inaccuracy and loss of discrimination, even though the detection of carcinogens is improved. All the tests were shown to generate both false positive and false negative results, a situation which may be controlled by the use, where possible, of appropriate chemical-class controls, to identify the test which is optimal for the class of chemical under test. Structural analogy may have a part to play in the rapid detection of environmental carcinogens, and some general guidelines for its use are given.


*Br. J. Cancer* (1978) **37**, 873

### AN EVALUATION OF 6 SHORT-TERM TESTS FOR DETECTING ORGANIC CHEMICAL CARCINOGENS

I. F. H. PURCHASE, E. LONGSTAFF, JOHN ASHBY, J. A. STYLES, D. ANDERSON, P. A. LEFEVRE AND F. R. WESTWOOD

*From Imperial Chemical Industries Limited, Central Toxicology Laboratory, Alderley Park, Cheshire*

Received 2 November 1977 Accepted 6 February 1978

**Summary.—A number of tests have been described which are thought to be capable of identifying carcinogens without using the actual induction of cancer as an end-point. This study compares the performance of 6 such tests on a selection of 120 organic chemicals. The tests studies were: (1) mutation of *Salmonella typhimurium;* (2) cell transformation; (3) degranulation of endoplasmic reticulum; (4) sebaceous gland suppression; (5) tetrazolium reduction and (6) subcutaneous implant. A further 4 tests were examined briefly, but were not included in the complete evaluation.**

**The chemicals were classified into carcinogens (58) and non-carcinogens (62) on the basis of published experimental data, and into 1 of 4 broad chemical classes.**

**There was considerable variation between tests in their ability to predict carcinogenicity, with the cell-transformation test and the bacterial-mutation test being the most accurate (94% and 93% accurate respectively). These 2 tests were considered to be of general use in screening, since they were clearly more accurate than the others. Statistical consideration of various combinations of these tests showed that the use of cell transformation and bacterial mutation together, provide an advantage over the use of either test alone. The inclusion of the other 4 tests in a screening battery predictably resulted in a great increase in overall inaccuracy and loss of discrimination, even though the detection of carcinogens is improved. All the tests were shown to generate both false positive and false negative results, a situation which may be controlled by the use, where possible, of appropriate chemical-class controls, to identify the test which is optimal for the class of chemical under test. Structural analogy may have a part to play in the rapid detection of environmental carcinogens, and some general guidelines for its use are given.**

INFORMATION about the carcinogenic activity of chemicals has been gathered from epidemiological studies and animal experimentation. Epidemiological studies based on geographical variations in cancer incidence have indicated that many human cancers are caused, mediated or modified by environmental factors (Higginson, 1969; Boyland, 1969; Wynder and Mabuchi, 1972; Higginson and Muir, 1973; Cairns, 1975). Although one of the factors is thought to be the presence of naturally occurring and man-made carcinogens in the environment (Clayson, 1962; Hueper and Conway, 1964; Boyland, 1969; Dinman, 1974; Weisburger and Williams 1975) those human cancers which are *known* to be caused by chemicals are few in number. These cancers, which have been associated with specific chemicals have generally been limited to groups of people in a particular industry or occupation and, in total, make up only a small part of the cancer burden in man. Because of the retrospective nature of epidemiology, a carcinogenic hazard cannot be anticipated. Furthermore, epidemiological studies are expensive and time-consuming, and the gathering of complete and statistically analysable data is difficult.

Many hundreds of other chemicals, however, have been shown to be carcinogenic in animals (WHO/IARC publications 1972–75) and are, therefore, potentially carcinogenic to man. Carcinogenesis studies in animals can be used at present to identify potential human carcinogens, but suffer from problems of interpretation due to modifying factors such as diet, variations in spontaneous and induced tumour incidences, species, strain and sex differences. Further disadvantages of animal testing are high costs, protracted duration of such studies and the resultant heavy demands on animals and laboratory resources.

There are many thousands of environmental and industrial chemicals and to test every chemical for carcinogenic activity in animals would obviously be very expensive and impracticable within the foreseeable future. It is for this reason that attempts are being made to develop short term tests with non-cancerous end points to identify carcinogenic chemicals.

There is now a greater understanding of some of the mechanisms involved in chemical carcinogenesis from metabolic and structure-activity correlation studies (Clayson, 1962; Brookes, 1971; Hueper and Conway, 1964; WHO/IARC, 1974; Miller, 1970; Miller and Miller, 1971*a*, *b*, 1972, 1974; Dinman, 1974; Arcos and Argus, 1974) but in no case is there unequivocal knowledge of the molecular target critical to the induction of cancer (Miller, 1970).

Tests having non-cancerous end-points were often derived from observations on the effects produced by carcinogens, and were adapted to screen chemicals (Montesano *et al.*, 1976; Stoltz *et al.*, 1974; Brookes and de Serres, 1976; Bridges, 1976). Their chief advantages are rapidity, low cost and simplicity of operation, thereby enabling a large number of chemicals to be tested.

The main disadvantage of any test with a non-cancerous end-point is that the significance of the test response with regard to carcinogenicity must be carefully assessed. With the exception of the Ames' test (McCann *et al.*, 1975) no extensive evaluation has been made of any of these tests. Furthermore with the exception of a few reviews (Stoltz *et al.*, 1974; Brookes and de Serres, 1976) no attempt has been made to compare tests.

We consider that detailed examination of the available tests (Stoltz *et al.*, 1974; Montesano *et al.*, 1976; Brookes and de Serres, 1976) for predicting carcinogenicity was impracticable. Instead, several tests were selected for evaluation, bearing in mind the available expertise, facilities and published confidence in the various test procedures. The methods selected for study were the following:

(1) Ames test. *Salmonella typhimurium* plate-incoporation mutagenicity assay (Ames *et al.*, 1975).
(2) Cell transformation. Mammalian cell transformation in culture (Styles, 1977).
(3) Rabin's test. Degranulation of rough endoplasmic reticulum from rat liver (Williams and Rabin, 1971).
(4) Sebaceous-gland test. Mouse-sebaceous-gland suppression (Bock and Mund, 1958),
(5) Tetrazolium-reduction test. Reduction of tetrazolium red by mouse skin (Iversen and Evensen, 1962).
(6) Implant test. Tissue reaction to subcutaneous implants in mice (Westwood and Longstaff, unpublished).

A further 4 tests were considered, but after a brief evaluation were found to be unsuitable (see Appendix VIII).

The 120 chemicals chosen for the validation study were selected from a variety of structural and functional classes, and consisted of 58 carcinogens and 62 non-carcinogens. This paper reports the results of a comparative evaluation carried out on a number of short-term tests purporting to identify chemical carcinogens. The evaluations were also aimed at comparing the tests individually and in groups, in order to arrive at the most useful combination.

A preliminary report has already been published (Purchase *et al.*, 1976).

### MATERIALS AND METHODS

### *Criteria used to classify substances tested for carcinogenicity*

*Category of carcinogenicity.*—Only 2 categories of carcinogenicity have been used, namely carcinogenic or non-carcinogenic. Compounds have been classified by an assessment of the available scientific literature wherever possible. The assessment criteria were as follows:

(a) Any materials shown to produce malignant tumours in any mammalian species as a result of application to the skin, i.p. or i.v. injection, or orally (including intragastrically), have been regarded as carcinogenic.

(b) Initiating and promoting agents have been classified as carcinogens.

(c) Tumours arising in the urinary bladder as a result of bladder implantation techniques have not been considered as meaningful.

(d) Tumours arising at the site of subcutaneous injection (*i.e.* as sarcomas) have been ignored unless accompanied by the appearance of tumours at other sites.

(e) Negative results after s.c. injection or bladder implantation have been regarded as significant, and were considered an indication of non-carcinogenesis.

(f) Where there is only an increase in the incidence of common tumours in mice (*e.g.* of hepatomas or lung adenomas in susceptible strains) the data have been ignored, unless there have been concurrent appearances of other tumours at different sites.

(g) Evidence based solely on the appearance of benign tumours has been considered insufficient for a positive classification.

(h) Compounds which were negative in studies which have continued for the major part of the animal's lifespan have been classified as non-carcinogens. Where there is no reason to suspect carcinogenicity (*e.g.* natural products of mammalian systems) or on theoretical grounds (*e.g.* by analogy with a closely related chemical known to be positive) then the compounds in question have been classed as negative.

*Chemical class.*—The compounds used in this study were selected to represent a wide range of carcinogens and non-carcinogens and have been somewhat arbitrarily sub-divided into the 4 chemical classes shown below. Where possible, structurally related carcinogen and non-carcinogen pairs were included. The compounds were coded, and each was tested without operator knowledge of their biological activity. The 4 main classes of chemicals are:

(a) *Polycyclics* (P). The group comprises polycyclic and heteropolycyclic aromatic compounds containing at least 2 fused aromatic rings. The group includes several substituted nuclei, but polycyclics bearing amino substituents have been excluded, as their carcinogenic metabolic activation is thought to be dominated by this substituent rather than the aromatic nucleus *per se*.

(b) *Alkylating Agents* (Alk). A variety of chemical classes are included in this group, all of which are capable of direct interaction with nucleophiles, giving alkylation products. Several alkyl nitrosamines are included, as they can give rise to alkylating species following suitable metabolic conversion.

(c) *Aromatic Amines* (AA). This group consists of various nuclei each substituted with an aromatic amino (anilino) or substituted amino group, which in the case of the active examples, dominates the carcinogenic response. The 2 groups of carcinogens centred on 4-aminobiphenyl and benzidine and the closely related nitrobiphenyls are also included in this group.

(d) *Miscellaneous* (M). This group contains compounds which do not obviously fit into any of the above groups on either a structural or a functional basis.

A list of these chemicals together with their group classification is given in Table I. The compounds were tested on only one occasion in each of the tests, except where additional results are mentioned. This was a deliberate decision and does not reflect routine laboratory practice where experiments may be repeated. Compounds which gave incorrect results were not re-tested; for example, the incorrect negative results on vinyl chloride, which is reproducibly positive in the Ames test when tested as a gas in this laboratory, was not altered during the validation study.

The effect of testing on only one occasion would tend to reduce the accuracy of the tests. One consequence of this approach, however, is that too much emphasis should not be placed on individual results reported in Table I without repeating them.

*Short-term testing procedures*

*Chemical-structure correlation.*—Since the carcinogenic activities of the compounds used in this study were classified from a study of published information, chemical-structure correlation was not used. However, when tests are in routine use, a knowledge of the structure of the compound would enable analogies to be drawn between the test compound and known carcinogens and non-carcinogens. This would have 2 purposes: firstly, to act as a primary screen to select those compounds most worthy of attention; and secondly, as a means of monitoring the results of rapid screening, which will always have a predictive accuracy of less than 100%. One way in which chemical-structure correlation may be used to predict carcinogenic activity is given in Appendix I.

*Bacterial mutation.*—Compounds were tested using 4 strains of Salmonella in an assay medium containing rat liver post-mitochondrial supernatant and cofactors (S-9 mix) according to the method of Ames *et al.* (1975). Details of the method are given in Appendix II.

*Mammalian-cell transformation in culture.*—A new technique was developed in which Syrian hamster kidney cells (BHK 21/cl 13) and either human diploid lung fibroblasts (W1-38) or human liver derived cells (Chang) were exposed to 5 different doses of the test compounds *in vitro* in serum-free liquid tissue-culture medium containing rat post-mitochondrial supernatant and cofactors (S-9 mix: Ames *et al.*, 1975) to aid in metabolism of the test compound. Following incubation, cells were centrifuged and the medium containing the compound and microsomes discarded. The cells were resuspended in growth medium. To assess survival following exposure an aliquot (10 $\mu$l) containing about 1000 cells was incubated in liquid medium and colonies counted after 6–8 days. A dose-response curve for survival was constructed and the $LC_{50}$ calculated. To the remaining cell suspension was added molten agar to give a concentration of 0·3% agar in medium, which allowed the growth of transformed colonies (Macpherson and Montagnier, 1964). Colonies were counted after 3 weeks' incubation, a dose-response curve for transformation constructed and the number of transformed colonies at the $LC_{50}$ calculated. A 2·5 times increase in colony numbers over controls was regarded as positive. Although this test is referred to as cell transformation, growth in semi-solid agar is only one of the accepted criteria for cell transformation. Details of the test method are given in Appendix III.

*Degranulation of rat liver rough endoplasmic reticulum.*—The loss of ribosomes from isolated rat liver endoplasmic reticulum (RER) following incubation with carcinogens *in vitro*, first described by Williams and Rabin (1971) has been quantitatively assayed by radio-tracer techniques (Purchase and Lefevre, 1975). A statistically significant increase in degranulation of the RER by the test compound, over negative controls was taken to indicate a positive result. The method is described in Appendix IV.

*Tetrazolium reduction.*—The test was based on that described by Iversen and Evensen (1962). Samples of mouse skin which had been exposed to the test compound *in vivo*, were incubated in aqueous solutions of tetrazolium red. Increases in *in situ* biological reduction of the colourless tetrazolium compound to the coloured formazan were measured spectrophotometrically, and taken to indicate a positive response for the test compound. The method is given in detail in Appendix V.

*Mouse-skin sebaceous-gland suppression.*—Bock and Mund (1958) have demonstrated that the sebaceous glands of mouse skin are sensitive to the topical application of carcinogens. Test chemicals were applied directly to mouse skin, and those causing a statistically significant depression of the ratio of sebaceous glands to hair follicles were taken to be positive. Details of this test are given in Appendix VI.

*Subcutaneous implant.*—A novel technique has been developed (Longstaff and Westwood, unpublished) based on the s.c. implantation in groups of mice of Millipore-filter discs overlaid with a gelatinous suspension of the test compound. The tissue surrounding the implant was examined histologically after the 3-month test period and the lesions scored on an arbitrary scale. A positive result was recorded when the group mean was significantly increased. The method is described in Appendix VII.

### RESULTS

The results obtained for each compound from the different tests in this study are given in Table I. Each compound in the list is followed by its source, chemical class, carcinogenicity and reference to animal studies. The remaining columns show the results of each test. Detailed tabulations of results from each test can be found in the appropriate Appendix, and the detailed results from the 8 (+, —) pairs of compounds in the cell transformation and bacterial mutation tests are given in Fig. 3–6.

Table II shows the number of compounds correctly identified by each of the short-term tests. The data are presented for each chemical class and for all compounds.

Table III gives the percentage correct predictions made by each test on the complete group of 120 compounds and on each chemical class. The figures in parentheses are corrected for equal numbers of carcinogens and non-carcinogens in each chemical class.

TABLE I.—*Summary of Short-term Predictive Tests for Carcinogenicity*

| Compound | Source | Chemical class and carcinogenicity (8) | Reference | Bacterial mutation | Cell transformation | Degranulation | Sebaceous-gl suppression | Tetrazolium reduction | Implant (9) |
|---|---|---|---|---|---|---|---|---|---|
| Acridine | D, G | P— | 1 | — | — | — | — | — | — |
| 2-Acetylaminofluorene | C | AA+ | 79, 80 | + | + | + | — | — | — |
| 4-Acetylaminofluorene | E | AA— | 81 | — | — | + | Nt | Nt | Nt (10) |
| Aflatoxin B | H | M+ | 2 | + | + | + | + | — | — |
| 4-Aminoazobenzene | H | AA+ | 3 | + | + | + | + | + | — |
| 2-Aminobiphenyl | B | AA+ | 4 | + | + | — | — | + | — |
| 4-Aminobiphenyl | H | AA+ | 5, 6, 7, 8 | + | + | + | — | — | + |
| 2-Aminochrysene | C, F | AA+ | 9 | + | + | + | + | — | ++ |
| 6-Aminochrysene | C | AA+ | 9 | + | + | + | + | + | + |
| 3-Aminopyrene | B | AA+ | 10 | + | + | + | — | + | + |
| 2-Aminonaphthalene-1-sulphonic acid | L | AA— | — | — | — | + | — | — | — |
| Aniline | D | AA— | 11, 12, 13 | — | — | — | — | — | — |
| p-Anisidine | B | AA— | — | — | — | + | + | — | — |
| Anthracene | D | P— | 14, 15 | — | — | + | — | — | — |
| 2-Aminoanthracene | B | AA+ | 16, 17 | + | + | + | + | — | — |
| Anthranilic acid | B | AA— | 18 | — | — | — | — | — | — |
| Anthraquinone | L | M— | 19 | — | — | — | + | — | — |
| Anthrone | D | M— | — | — | — | — | — | + | — |
| 1,2-Benzanthracene | D | P+ | 20, 21, 17 | + | + | + | + | + | — |
| Benzanthrone | A | M— | 22 | — | — | + | — | — | — |
| Benzidine | J | AA+ | 23 | + | + | + | + | — | — |
| Benzimidazole | F | P— | — | — | — | — | — | + | — |
| Benzoic acid | B | M— | — | — | — | — | — | — | — |
| 3,4-Benzpyrene | B, C | P+ | 24, 25, 26 | + | + | + | + | — | ++ |
| 6-Benzoyl-2-naphthol | B | M— | — | — | + | + | + | — | — |
| Biphenyl | A | M— | 19 | — | — | — | + | — | — |
| Bis azo compound (7) | L | M— | 97 | — | — | — | + | — | — |
| Bis(Chloromethyl)ether | D | Alk+ | 32 | + | + | — | + | + | + |
| N,N′-Bis(2-naphthyl)-p-phenylenediamine | L | AA— | 19 | — | — | + | — | — | + |
| Butanesultone | H | Alk+ | 27 | + | + | + | + | + | Nt |
| Caffeine | B | M— | 28 | — | — | — | + | — | — |
| Calmagite (1) | B | M— | — | — | — | + | + | — | — |
| Camphor | B | M— | 29, 30 | — | — | — | + | + | + |
| Carbazole | A | P— | — | — | — | + | — | — | — |
| Chlorambucil | B | Alk+ | 31 | + | + | — | + | — | Nt |
| Chloramine T | A | M— | — | — | — | — | — | — | — |
| Cholesterol | B | M— | 33 | — | — | — | — | — | — |

TABLE I.—*contd.*

| Compound | Source | Chemical class and carcinogenicity (8) | Reference | Bacterial mutation | Cell transformation | Degranulation | Sebaceous-gland suppression | Tetrazolium reduction | Implant (9) |
|---|---|---|---|---|---|---|---|---|---|
| Colchicine | B | M− | 20 | − | − | − | + | + | Nt |
| Croton oil | B | M+ | 37 | + | − | + | + | + | + |
| Cyanocobalamin (B12) | B | M− | — | − | − | − | − | + | − |
| Cycasin acetate (2) | S | Alk+ | 35 | + | + | + | + | − | − |
| Cyclohexylamine | B | M− | 36, 37 | − | − | + | + | − | − |
| Cyclophosphamide | C | Alk+ | 38 | + | + | + | − | − | + |
| 3,3′-Diaminobenzidine | L | AA− | 16 | + | − | + | + | − | − |
| 2,7-Diaminofluorene | B | AA+ | 39 | − | + | + | − | + | + |
| 3,4,5,6-Dibenzacridine | B, D | P+ | 42 | + | − | + | + | − | − |
| 1,2,3,4-Dibenzanthracene | B, C | P+ | 43 | + | + | − | + | − | ++ |
| 3,4,9,10-Dibenzpyrene | B, P | P+ | 44, 45 | + | + | + | + | + | ++ |
| 3,3′-Dichlorobenzidine | L | AA+ | 41, 46 | + | + | + | − | − | − |
| 2,4-Dichlorophenoxyacetate | A | M− | 19, 47 | − | − | + | − | − | − |
| Dicyclohexylamine | L | M− | 36 | − | − | − | − | + | − |
| D.D.T. (3) | A | M− | 48, 49, 50, 51 | − | − | − | − | − | − |
| Dieldrin (4) | D | M− | 52, 53 | − | − | − | − | Nt | − |
| Diethylnitrosamine | R | Alk+ | 54, 55, 56 | + | + | + | − | + | − |
| Diethylstilboestrol | B | M+ | 57, 58 | − | − | − | − | + | − |
| 3,3′-Dimethoxybenzidine | A, B | AA+ | 40, 41 | + | + | + | + | − | − |
| 4-Dimethylaminoazobenzene | D | AA+ | 59, 60 | + | + | + | − | + | + |
| 9,10-Dimethylanthracene | B | P+ | 61 | + | + | − | + | + | + |
| p-Dimethylaminobenzaldehyde | D | AA− | — | − | − | − | − | − | − |
| 7,9-Dimethylbenzacridine | B | P+ | — | + | + | + | + | − | Nt |
| 7,10-Dimethylbenzacridine | B, C | P+ | — | + | + | + | + | − | Nt |
| 9,10-Dimethyl-1,2-benzanthracene | B, D | P+ | 62, 63, 64 | + | + | + | + | + | ++ |
| 1,1′-Dimethyl-4,4′-bipyridinium dichloride | M | M− | 106, 107 | − | − | − | + | − | − |
| 3,3′-Dimethylbenzidine | D | AA+ | 114 | + | + | + | + | − | − |
| Dimethylcarbamoyl chloride | D | Alk+ | 65 | + | + | − | + | − | ++ |
| Dimethylformamide | A, B | M− | 66, 67, 68 | − | − | − | − | − | − |
| Dimethylnitrosamine | R | Alk+ | 69, 70, 71 | − | + | + | − | + | − |
| 2,3-Dimethylquinoxaline | L | P− | — | − | − | + | + | − | − |
| Dinitrobenzene | C, D | M− | — | − | − | − | + | − | − |
| 2,4-Dinitrofluorobenzene | D | M+ | 72 | + | + | − | + | − | − |
| 2,4-Dinitrophenol | B, D | M− | 73 | − | − | + | − | − | − |
| Dinitrosopentamethylene tetramine | L | M− | 40 | − | − | − | − | − | − |
| DL-Ethionine | A, B, C | Alk+ | 75 | − | + | + | − | + | − |
| 1,1′-Ethylene-2,2′-bipyridinium dibromide | M | M− | 74 | − | − | − | + | − | − |
| Ethylenethiourea | L | M+ | 19, 76 | + | + | − | − | − | − |
| Ethyl methanesulphonate | C, F | Alk+ | 77, 78 | + | + | − | + | − | − |
| Hexachlorocyclohexane | A | M− | 48, 82 | − | − | + | − | + | − |
| Hexamethylphosphoramide | A | M+ | 83 | + | + | − | − | − | − |
| Hydrazine | K | M+ | 84 | + | + | − | + | − | − |
| Hydrocortisone | B | M− | — | − | + | − | + | − | − |
| Indole | B | P− | 85, 86, 87, 88 | − | − | − | + | + | − |
| Merchlorethamine (5) | B, N | Alk+ | 31, 89, 90 | + | + | − | + | − | − |
| 20-Methylcholanthrene | B, C, D | P+ | 91, 92, 93 | + | + | + | + | + | ++ |
| Methylene bis(2-chloroaniline) | L | AA+ | 46 | + | + | + | + | − | − |
| 2-Methylindole | B, D | P− | — | − | − | + | − | + | − |
| MNNG (6) | C | Alk+ | 94, 95 | + | + | + | + | + | − |
| 3-Methyl-4-nitroquinoline-N-oxide | T | AA− | 96 | − | − | − | − | − | − |
| Mitomycin C | B | Alk+ | — | + | + | + | + | − | − |
| Morgan's base | B | P+ | 42 | + | + | − | + | − | Nt |
| Naphthalene | D | P− | 15 | − | − | − | − | − | − |
| 1-Naphthol | D | M− | 98 | + | − | − | − | − | − |
| 2-Naphthol | D | M− | 98 | − | − | − | − | + | − |

TABLE I.—*contd.*

| Compound | Source | Chemical class and carcinogenicity (8) | Reference | Bacterial mutation | Cell transformation | Degranulation | Sebaceous-gland suppression | Tetrazolium reduction | Implant (9) |
|---|---|---|---|---|---|---|---|---|---|
| 1-Naphthylamine | B | AA− | 99, 41, 13 | − | − | − | − | − | − |
| 2-Naphthylamine | L | AA+ | 100, 41, 13 | + | − | + | + | + | − |
| 2-Naphthylamine-1,5-disulphonic acid disodium salt | L | AA− | — | − | − | − | − | + | − |
| Nitrobenzene | L | M− | — | − | − | − | − | + | − |
| 2-Nitrobiphenyl | C, D | AA+ | 101 | + | + | + | − | − | Nt |
| 4-Nitrobiphenyl | B, D | AA+ | 101 | + | + | + | + | − | − |
| 2-Nitrofluorene | H | AA+ | 10 | + | + | + | − | − | Nt |
| N-Nitrosodiphenylamine | L | M− | 19 | − | − | − | + | + | − |
| N-Nitrosoephedrine | U | Alk+ | 102 | + | + | + | + | − | − |
| N-Nitrosofolic acid | U | Alk+ | 102 | + | + | − | − | − | − |
| 4-Nitroquinoline-N-oxide | L | AA+ | 103 | + | + | + | + | + | + |
| 4-Nonylphenol/ethylene oxide condensate | L | M− | — | − | − | − | − | − | − |
| Orotic acid | B | M− | 105 | − | − | − | + | − | − |
| Perylene | B, C | P− | 108 | + | − | − | − | + | − |
| Phenobarbital | B | M− | 48, 28 | − | − | − | + | + | − |
| N-phenyl-2-naphthylamine | L | AA− | 19, 104 | − | − | + | + | + | − |
| Propanesultone | L | Alk+ | 109 | + | + | + | − | + | + |
| β-Propiolactone | B, C | Alk+ | 110 | + | + | + | + | − | − |
| Resorcinol | A | M− | — | − | − | − | − | − | − |
| Riboflavin | B | M− | 111 | − | − | − | − | − | − |
| Safrole | H | M+ | 112 | + | + | + | + | − | − |
| 3,3′,5,5′-Tetramethylbenzidine | U | AA− | 113 | − | − | + | − | − | − |
| Toluene | A | M− | 115 | − | − | − | − | − | − |
| Toluene-2,4-diisocyanate | L | M− | — | − | − | − | − | − | + |
| 2,4,5-Trichlorophenoxyacetate | A | M− | 19 | − | − | − | + | − | − |
| Trimethylphosphate | A | M− | 116 | + | − | − | − | − | Nt |
| Urethane | A, B | M+ | 117, 118 119 | + | + | − | − | + | + |
| Vinyl chloride | E | Alk+ | 120, 121 | − | − | − | + | − | − |

(1) Calmagite: 2-hydroxy-1-(2-hydroxy-5-methylphenylazo) naphthalene-4-sulphonic acid.
(2) Cycasin (acetate): methyloxymethanol acetate.
(3) D.D.T.: 1,1,1-trichloro-2,2-bis(4-chlorophenyl)ethane.
(4) Dieldrin: 1,2,3,4,10,10-hexachloro-6,7-epoxy-1,4,4a,5,6,7,8,8a-octahydro-endo-1,4-exo-5,8-dimethanonaphthalene.
(5) Merchlorethamine: bis(2-chloroethyl)methylamine hydrochloride.
(6) MNNG: N-methyl-N′-nitro-N-nitrosoguanidine.
(7) Bis azo compound: 2,2′-Bis[1-(3-octadecylaminopropylimino)ethyl]-2,2′-[3,3′-dichloro-4,4′-biphenylylene)bis(azo)]bis(acetanilide).
(8) Chemical class and carcinogenicity:
AA Arylamines and related compounds.
Alk Alkylating agents.
P Polycyclic aromatic hydrocarbons.
M Miscellaneous compounds.
\+ Carcinogen.
− Non-carcinogen.
(9) In the results of the implant test, ++ denotes presence of tumour at site of implant.
(10) Nt: not tested.

*Source of chemicals*

A. BDH Chemicals, Shaw Road, Speke, Liverpool.
B. Sigma (London) Chemical Co., Ltd, Norbiton Station Yard, Kingston upon Thames.
C. Koch-Light Laboratories, Colnbrook, Bucks.
D. Fluorochem Ltd (Fluka), Dinting Vale Trading Estate, Glossop, Derbys.
E. Air Products Ltd, Sharp Street, Worsley, Walkden, Lancs.
F. Phase Separations Ltd, Deeside Industrial Estate, Queensferry, Flints.
G. Ralph N. Emmanuel Ltd, 264 Water Road, Alperton, Middx.
H. Aldrich Chemicals, Old Brick Yard, New Road, Gillingham, Dorset.

J. May and Baker, Dagenham, Essex.
K. Hopkin and Williams Ltd, The Laboratory Centre, Ducie Street, Manchester.
L. ICI Ltd, Organics Division, Hexagon House, Blackley, Manchester.
M. ICI Ltd, Plant Protection Division, Fernhurst, Hazelmere, Surrey.
N. The Boots Company Ltd, Nottingham.
P. K. & K. Ltd, Kodak Ltd, Kirkby, Liverpool.
R. Eastman Ltd, Kodak Ltd, Kirkby, Liverpool.
S. Schwartz Mann, Uniscience Ltd, Airfleet House, Sullivan Road, London.
T. Lancaster Synthesis Ltd, St Leonards House, St Leonardgate, Lancaster.
U. J. Ashby and D. Paton, ICI Central Toxicology Laboratory.

*References to Carcinogenic Studies* (*Table* 1)

1. SHUBIK, P. (1949) Studies on the promoting phase in the stages of carcinogenesis in mice, rats, rabbits and guinea pigs. *Cancer Res.*, **9**, 13.
2. BUTLER, W. M. & BARNES, J. M. (1968) Carcinogenic action of ground-nut meal containing aflatoxin in rats. *Fd. Cos. Toxicol.*, **6**, 135.
3. KIRBY, A. H. M. (1947) Studies with Carcinogenesis with azo compounds III. The action of (A) Four azo compounds in Wistar rats fed restricted diets: (B) N,N-Diethyl-p-Aminoazobenzene in mice. *Cancer Res.*, **7**, 333.
4. MILLER, E. C., MILLER, J. A., SANDIN, R. B. & RUSCH, H. P. (1956) The carcinogenicity of compounds related to 2-acetylaminoflourene. III. Aminobiphenyl and benzidine derivatives. *Cancer Res.*, **16**, 525.
5. CLAYSON, D. B., LAWSON, T. A. & PRINGLE, J. A. S. (1967) The carcinognic actions of 2-aminodiphenylene oxide and 4-aminodiphenyl on the bladder and liver of C57X1F mouse. *Br. J. Cancer*, **21**, 755.
6. MILLER, E. C. FLETCHER, T. L., MARGRATH, A. & MILLER, J. A. (1962) The carcinogenicities of derivatives of flourine and biphenyl. Fluoro derivatives as probes for active sites in 2-acetylaminofluorene. *Cancer Res.*, **22**, 1002.
7. BONSER, G. M. (1962) Pre-cancerous changes in the urinary bladder. In *The Morphological Precursor of Cancer*. Ed. Servi, L. Perugia. p. 435.
8. WALPOLE, A. L., WILLIAMS, M. H. C. & ROBERTS, D. C. (1954) Tumours of the urinary bladder in dogs after ingestion of 4-aminodiphenyl. *Br. J. indust. Med.*, **11**, 105.
9. LAMBELIN, G., ROBA, J., RONCUCCI, R. & PARTMENTIER, R. (1975) Carcinogenicity of 6-aminochrysene in mice. *Eur. J. Cancer*, **11**, 327.
10. MILLER, J. A., SANDIA, R. B., MILLER, E. C. & RUSCH, H. P. (1955) The carcinogenicity of compounds related to 2-acetylaminofluorene II. Variations in the bridges and the 2-substituent. *Cancer Res.*, **15**, 188.
11. DRUCKERY, H. (1950) Beitrage zur Pharmakologie cancerogener Substanzen versuche mit anilin. *Arch. expl. Path. Pharmacol.*, **210**, 137.
12. BERENBLUM, I. & BONSER, G. M. (1937) Experimental investigation of "aniline cancer". *J. indust. Hyg. and Toxicol.*, **19**, 86.
13. GEHRMANN, G. H. FOULGER, J. H. & FLEMING, A. J. (1949) Occupational carcinoma of the bladder. *Proc. 9th Int. Congress Ind. Med.* London: Wright. p472.
14. SALAMAN, M. H. & ROE, F. J. C. (1956) Further tests for tumour-initiating activity: N,N-Di-(2-chloroethyl)-p-aminophenylburric acid (cb 1348) as an initiator of skin tumour formation in the mouse. *Br. J. Cancer*, **10**, 363.
15. SCHMAHL, D. (1955) Prufung von Naphthalin and Anthracen auf cancerogene Wirkung an Ratten. *Z. Krebsforsch;* **60**, 697.
16. GRISWOLD, D. P., CASEY, A. E., WEISBURGER, E. K. & WEISBURGER, J. H. (1968) The carcinogenicity of multiple intragastric doses of aromatic and heterocyclic nitro or amino derivatives in young female Sprague–Dawley rats. *Cancer Res.*, **28**, 924.
17. SHUBIK, P., PIETRA, G. & PORTA, G. D. (1960) Studies of skin carcinogenesis in the Syrian Golden Hamster. *Cancer Res.*, **20**, 100.
18. ECKMAN & STROMBECK (1949) The effect of some split products of 2,3′-azotoluene on the urinary bladder in the rat and their excretion on various diets. *Acta Pathol. Micro Scand.*, **26**, 447.
19. INNES, J. R. M., ULLAND, B. M., VALERIO, M. G., PETRUCELLI, L., FISHBEIN, L., HART, E. R., PALLOTTA, A. J., BATES, R. R., FALK, H. L., GART, J. J., KLEIN, M., MITCHELL, I. & PETERS, J. (1969) Bioassay of pesticides and industrial chemicals for tumourigenicity in mice: A preliminary note. *J. natn. Cancer Inst.*, **42**, 1101.
20. ROE, F. J. C. & SALAMAN M. H. (1955) Further studies on incomplete carcinogenesis: Triethylene melamine (TEM) 1,2-benzanthracene and β-propiolactone, as initiators of skin tumour formation in the mouse. *Br. J. Cancer*, **9**, 177.
21. PATAKI, J. & HUGGINS, C. (1969) Molecular site of substituents of benz(a)anthracene related to carcinogenicity. *Cancer Res.*, **29**, 506.
22. MOROSENSKAYA, S. (1940) Referred to in *Survey of compounds which have been tested for carcinogenic activity*. PHS publication No. 149 (1951), 542.
23. BOYLAND, E., HARRIS, J. & HORNINGS, E. S. (1954) The induction of carcinoma of the bladder in rats with acetamidoflourene. *Br. J. Cancer*, **8**, 647.
24. SHUBIK, P. & DELLA PORTA, G. (1957) Carcinogenesis and acute intoxication with large doses of polycyclic hydrocarbons. *Archs. Path.*, **64**, 691.
25. HUGGINS, C. & YANG, N. C. (1962) Induction and extinction of mammary cancer. *Science*, **137**. 257.
26. CHU, E. W. & MALMGREN, R. A. (1968) The inhibitory effect of Vit. A on the induction of tumours of forestomach and cervix in the Syrian hamster by carcinogenic polycyclic hydrocarbons. *Cancer Res.*, **25**, 884.

27. Druckrey, H., Kruse, H., Preussmann, R., Ivankovic, S., Landschutz, C. & Gimmy, J. (1970) Cancerogenic alkylating substances IV. 1,3-Propane sultone and 1,4-butane sultone. *Z. Krebsforsch*, **75**, 69.
28. Boughton, L. L. & Stoland, O. O. (1943) The effect on estrus of drugs administered daily in therapeutic doses throughout the life cycle of albino rats and the estrus cycle sequence with reference to age. *J. Am. Pharm. Ass.*, **32**, 187.
29. Stoner, G. D., Shimkin, M. B., Kniazeff, A. J. Weisburger, E. K., & Gori, G. B. (1973) Test for carcinogenicity of food additives and chemotherapeutic agents by the pulmonary tumour response in Strain A mice. *Cancer Res.*, **33**, 3069.
30. Ezeyza, S. (1952) Carencia de Poder Cancerigeno del Guayacol Alcaufor y Ruibarbo e Intensa Accion Irritativa del Ultimo, Probados Subcutaneamente en Ratas. *Semana Med.*, **100**, 663.
31. Shimkim, M. B., Weisburger, J. H., Weisburger, E. K., Gubareff, N. & Suntzeff, V. (1966) Bioassay of 29 alkylating chemicals by the pulmonary tumour response in strain A mice. *J. natn. Cancer Inst.*, **36**, 915.
32. Laskin, S., Kuschner, M., Drew, T. R., Cappiello, V. P. & Nelson, N. (1971) Tumours of the respiratory tract induced by inhalation of bis(chloromethyl)ether. *Arch. environ. Health*, **23**, 135.
33. Bischoff, F. & Bryson, G. (1964) Carcinogenesis through solid state surfaces. *Prog. exp. Tumour Res.*, **5**, 85.
34. Roe, F. J. C. & Salaman, M. H. (1955) Further studies on incomplete carcinogenesis; triethylene melamine (TEM), 12,-benzanthracene and β-propiolactone, as initiators of skin tumour formation. *Br. J. Cancer*, **9**, 177.
35. Lacqueur, G. L. (1965) The induction of intestinal neoplasms in rats with the glycoside cycasin and its aglycone. *Virchows Arch. path. Anat.*, **340**, 151.
36. Pliss, G. B. (1958) On the carcinogenic activity of dicyclohexylamine and dicyclohexylamine nitrite. *Vop. Onkol.*, **4**, 659.
37. Price, J. M., Biava, C. G., Oser, B. L., Vogin, E. E., Steinfeld, J. & Ley, H. L. (1970) Bladder tumours in rats fed cyclohexylamine or high doses of a mixture of cyclamate and saccharine. *Science*, **167**, 1131.
38. Weisburger, E. K. (1975) A critical evaluation of the methods used for determining carcinogenicity. *J. Clin. Pharm.*, **5**, 5.
39. Morris, H. P., Wagner, B. P., Ray, F. E., Stewart, H. L. & Snell, K. C. (1963) Carcinogenic effects of N,N′-2,7-fluorenylene bis-2,2,2-Trifluoroacetamide (2,7-FAA-FA6) administered orally to Buffalo strain rats. *J. natn. Cancer Inst.*, **30**, 143.
40. Hadidian, Z., Fredrickson, T. N., Weisburger, E. K., Weisburger, J. H., Glass, R. M. & Mantel, N. (1968) Tests for chemical carcinogens. Report on the abilityof derivatives of aromatic amines, nitrosamines, quinolines, nitroalkanes, amides, epoxides, aziridines and purine antimetabolites. *J. natn. Cancer. Inst.*, **41**, 985.
41. Sellakumar, A. R., Montesano, R. & Saffiotti, U. (1969) Aromatic amines carcinogenicity in hamsters. *Proc. Am. Ass. Cancer Res.*, **10**, 78.
42. Wynder, E. L. & Hoffmann, D. (1963) Einexperimenteller Beitrag zur Tabakrauchkanzerogenese. *D. med. Wschr.*, **88**, 623.
43. Lijinsky, W., Garcia, H. & Saffiotti, U. (1970) Structure-activity relationships among some polynuclear hydrocarbons and their hydrogenated derivatives. *J. natn. Cancer Inst;* **44**, 641.
44. Homberger, F. & Tregier, A. (1960) Modifying factors in carcinogenesis. *Prog. exp. Tumour Res.*, **1**, 311.
45. Sellakumar, A. & Shubik, P. (1974) Carcinogenicity of different polycyclic hydrocarbons in the respiratory tract of hamsters. *J. natn. Cancer Inst.*, **53**, 1713.
46. Stula, E. F., Sherman, H. & Zapp, J. A. (1953) Experimental neoplasia in CLR-CD rats with the oral administration of 3,3′-dichlorobenzidine, 4,4′-methylene bis (2-chloroaniline), and 4,4′-methylene bis (2-methylaniline). *Tox. appl. Pharmacol.*, **19**, 380.
47. Hansen, W. H., Quaife, M. L., Habermann, R. T. & Fitzhugh, O. G. (1971) Chronic toxicity of 2,4-dichlorophenoxyacetic acid in rats and dogs. *Tox. appl. Pharmacol.*, **20**, 122.
48. Thorpe, E. & Walker, A. I. T. (1973) The toxicity of dieldrin (HEOD) II. Comparative long-term oral toxicity studies in mice with dieldrin, DDT, phenobarbitone, β-BHC and γ-BHC. *Fd Cosmet. Toxicol.*, **11**, 433.
49. Kimbrough, R., Gaines, T. B. & Sherman, J. D. (1964) Nutritional factors long-term DDT intake and chloroleukemia in rats. *J. natn. Cancer Inst.*, **33**, 215.
50. Agthe, C., Garcia, H., Shubik, P., Tomatis L. & Wenyon, E. (1970) Study of the potential carcinogenicity of DDT in Syrian golden hamsters. *Proc. Soc. exp. Biol. Med.*, **134**, 113.
51. Lehman, A. J. Ed. (1965) DDT (a mixture of 1,1,1-trichloro-2,2-bis (p-chlorophenyl) ethane and 1,1,1-trichloro-2-(o-chlorophenyl)-2-(p-chloro-phenyl)ethane). In *Summaries of Pesticide Toxicity*, FDA, U.S. Dept. Health, Education and Welfare, Washington, DC. p. 17.
52. Walker, A. I. T., Thorpe, E. & Stevenson, D. E. (1973) The toxicity of dieldrin (HEOD) I. Long term oral toxicity studies in mice. *Fd Cosmet. Toxicol.*, **11**, 415.
53. Walker, A. I. T., Stevenson, D. E., Robinson J., Thorpe, E. & Roberts, M. (1969) The toxicity and pharmacodynamics of dieldrin (HEOD): two year oral exposures of rats and dogs. *Tox. appl. Pharmacol.*, **15**, 345.
54. Clapp, N. K., Craig, A. W. & Toya R. E. (1970) Diethylnitrosamine oncogenesis in RF mice as influenced by variations in cumulative dose. *Int. J. Cancer*, **5**, 119.
55. Thomas, C. & Bollman, R. (1968) Investigations of trans-placental cancerogenic activity of diethylnitrosamine in rats. *Z. Krebsforsch*, **71**, 129.
56. Herrold, K. McD. (1964) Effect of route of administration on the carcinogenic action of diethylnitrosamine (N-nitrosodiethylamine). *Br. J. Cancer*, **18**, 763.
57. Dunning, W. F., Curtis, M. R. & Segaloff, A. (1947) Strain differences in response to diethylstilboestrol and the induction of mammary gland and bladder cancer in the rat. *Cancer Res;* **7**, 511.
58. HOLLAND, J. M. & HYATT, C. (1969) Urinary lactic dehydrogenase levels in experimental renal carcinogenics. *Invest. Virol.*, **6**, 631.
59. AKAMATSU, Y. & IKEGAMI, R. (1968) Induction of hepatoma and systemic amyloidosis in mice by 4-dimethylaminoazobenzene feeding. *Gann*, **59**, 201.
60. TAKAYAMA, S. & IMAIZUMI, T. (1969) Sequential effects of chemically different carcinogens, dimethylnitrosamine and 4-dimethylamino-azobenzene, on hepatocarcinogenesis in rats. *Int. J. Cancer*, **4**, 373.
61. LIJINSKY, W. & SAFFIOTTI, U. (1965) Relationships between structure and skin tumourigenic activity among hydrogenated derivatives of several polycyclic aromatic hydrocarbons. *Ann. Ital. Derm. Chem. Sper.*, **19**, 34.
62. ENGELBRETH-HOLM, J. & IVERSEN, S. (1951) On the mechanism of experimental carcinogenesis II. The effect of different concentrations of 9,10-dimethyl-1,2-benzanthracene on skin carcinogenesis in mice. *Acta Path. Microbiol. scand.*, **29**, 77.
63. GEYER, R. P., BLEISCH, V. R., BRYANT, J. E., ROBBINS, A. N., SASLOW, I. M. & STARE, F. J. (1951) Tumour production in rats injected intravenously with oil emulsions containing 9,10-dimethyl-1,2-benzanthracene. *Cancer Res.*, **11**, 474.
64. LEVY, B. M. & RING, J. R. (1950) Experimental production of jaw tumours in hamsters. *Oral Surg.*, **3**, 233.
65. VAN DUUREN, B. L., GOLDSCHMIDT, B. M., KATZ, C., SEIDMAN, I. & PAUL, J. S. (1974) Carcinogenic activity of alkylating agents. *J. natn. Cancer Inst.*, **53**, 695.
66. VAN DUUREN, B. L., MELCHIONNE, S., BLAIR, R., GOLDSCHMIDT, B. M. & KATZ, C. (1971) Carcinogenicity of isosters of epoxides and lactones: aziridine, ethanol, propane sultone and related compounds. *J. natn. Cancer Inst.*, **46**, 143.
67. CRADDOCK, V. M. (1971) Liver carcinomas induced in rats by single administration of dimethylnitrosamine after partial hepatectomy. *J. natn. Cancer Inst.*, **47**, 889.
68. HERROLD, K. McD. (1969) Aflatoxin induced lesions in Syrian hamsters. *Br. J. Cancer*, **23**, 655.
69. CLAPP, N. K., CRAIG, W. W. & TOYA, R. E. (1968) Pulmonary and hepatic oncogenesis during treatment of male RF mice with dimethylnitrosamine. *J. natn. Cancer Inst.*, **41**, 1213.
70. MAGEE, P. N. & BARNES, J. M. (1956) The production of malignant primary hepatic tumours in the rat by feeding dimethylnitrosamine. *Br. J. Cancer*, **10**, 114.
71. TOMATIS, L., MAGEE, P. N. & SHUBIK, P. (1964) Induction of liver tumours in the Syrian golden hamster by feeding dimethylnitrosamine. *J. natn. Cancer Inst.*, **33**, 341.
72. BOCK, F. G., FJELDE, A., FOX, H. W. & KELIN, E. (1969) Tumour promotion by 1-fluoro-2,4-dinitrobenzene, a potent skin sensitizer. *Cancer Res.*, **29**, 179.
73. SPENCER, H. C., ROWE, V. K., ADAMS, E. M. & IRISH, D. D. (1948) Toxicological studies on laboratory animals of certain alkyldinitrophenols used in agriculture. *J. Ind. Hyg. Toxicol.*. **30**, 10.
74. GOATER, T. O., KENYON, A. J. & WESTON-HURST, E. (1964) ICI report IHR/165.
75. SVOBODA, D. & HIGGINSON, J. (1968) A comparison of ultrastructural changes in rat liver due to chemical carcinogens *Cancer Res.*, **28**, 1703.
76. ULLAND, B. M., WEISBURGER, J. H., WEISBURGER, E. K., RICE, J. M. & CYPHER, R. (1972) Thyroid cancer in rats from ethylene thiourea intake. *J. natn. Cancer Inst.*, **49**, 583.
77. CLAPP, N. K. (1973) Carcinogenicity of nitrosamines and methanesulphonate esters given intraperitoneally in RF mice. *Int. J. Cancer*, **12**, 728.
78. HRUSHESKY, W., SAMPSON, D. & MURPHY, G. P. (1972) Carcinogenicity of ethylmethylsulphonate. *J. natn. Cancer Inst.*, **49**, 1077.
79. WOOD, M. (1969) Factors influencing the induction of tumours of the urinary bladder and liver by 2-acetylaminofluorene in the mouse. *Eur. J. Cancer*, **5**, 41.
80. ENGEL, R. W. & COPELAND, D. H. (1951) Influence of diet on the relative incidence of eye, mammary, ear-duct and liver tumours in rats fed 2-acetylaminofluorene. *Cancer Res.*, **11**, 180.
81. WEISBURGER, J. H., WEISBURGER, E. K. & MORRIS, H. P. (1952) Analogs of the carcinogen 2-acetylaminofluorene the isomeric 4-acetylaminofluorene. *J. Am. chem. Soc.*, **74**, 4540.
82. DAVIDOW, B. & FRAWLEY, J. P. (1951) Tissue distribution, accumulation and elimination of the isomers of benzene hexachloride. *Proc. Soc. exp. Biol. Med.*, **67**, 780.
83. ZAPP, J. A. (1975) Inhalation toxicity of hexamethylphosphoramide. *Am. ind. Hyg. Ass. J.*, **36**, 916.
84. SEVERI. L. & BIANCIFIORI, C. (1968) Hepatic carcinogenesis in CBA/Cb/Se mice and Cb/Se rats by isonicotinic acid hydrazide and hydrazine sulphate. *J. natn. Cancer Inst.*, **41**, 331.
85. ROE, F. J. C. & SALAMAN, M. H. (1955) Referred to in: *Survey of compounds which have been tested for carcinogenic activity.* PHS publication No. 149 suppl. 2 (1969) p. 494.
86. KAISER, K. (1953) Prufung des Indols auf cancergene Witkung bei Ratten. *Z. Krebsforsch.*, **59**, 488.
87. BOYLAND, E. & HORNING, E. S. (1949) Induction of tumours with nitrogen mustards. *Br. J. Cancer*, **3**, 118.
88. HESTON, W. E. (1953) Occurrence of tumours in mice injected subcutaneously with sulphur mustard and nitrogen mustard. *J. natn. Cancer Inst.*, **14**, 131.
89. HESTON, W. E. (1949) Induction of pulmonary tumours in strain A mice with methyl-bis ($\beta$-chloroethyl) amine hydrochloride. *J. natn. Cancer Inst.*, **10**, 125.
90. HESTON, W. E. (1950) Carcinogenic action of the mustards. *J. natn. Cancer Inst.*, **11**, 415.
91. FIRMINGER, H. I. & STEWART, H. L. (1951) Histopathogenesis of squamous cell carcinoma induced in the forestomach of mice by intramural injection of 20-methylcholanthrene. *J. natn. Cancer Inst.*, **12**, 491.
92. SHAY, H., HARRIS, C. & GRUENSTEIN, M.

(1951) Effect in male rats of the gastric instillation of methylcholanthrene in "heated" and "unheated" olive oil. *Cancer*, **4**, 988.
93. RUSSELL, W. O. & ORTEGA, L. R. (1952) Methylcholanthrene-induced tumours in guinea pigs. *Archs. Path.*, **53**, 301.
94. SCHOENTAL, R. & BENSTED, J. P. M. (1969) Gastrointesinal tumours in rats and mice following various routes of administration of N-methyl-N-nitroso-N′-nitroguanidine and N-ethyl-N-nitroso-N′-nitroguanidine. *Br. J. Cancer*, **23**, 757.
95. FUJIMURA, S., KOGURE, K., OBOSHI, S. & SUGIMURA, T. (1970) Production of tumours in glandular stomach of hamsters by N-methyl-N′-nitro-N-nitrosoguanidine. *Cancer Res.*, **30**, 1444.
96. HOSHINO, H., KAWAZOE, Y. & FUKUOKA, F. (1969) Detection of potential weak carcinogens and pre-carcinogens. I. Effect of the derivatives of 4-nitroquinoline 1-oxide on submanifestational of 4-nitroquinoline 1-oxide. *Gann*, **60**, 523.
97. CONNING, D. M. (1972) ICI report HO/IH/R 340.
98. SHEAR, M. J. & STEWART, H. L. (1941) In *Survey of compounds which have been tested for carcinogenic activity*. PHS Publication No. 149 (1951).
99. CLAYSON, D. B. & ASHTON, M. J. (1963) The Metabolism of 1-naphthylamine and its bearing on the mode of carcinogenesis of the aromatic amines. *Acta Un. Int. Cancer*, **19**, 539.
100. BONSER, G. M., CLAYSON, D. B., JULL, J. W. & PYRAH, L. N. (1952) The carcinogenic properties of 2-amino-1-naphthol hydrochloride and, its parent amine 2-naphthylamine. *Br. J. Cancer*, **6**, 412.
101. DEICHMANN, W. B., MACDONALD, W. M., CAPLAN, M. M., WOODS, F. M. & ANDERSON, W. A. D. (1958) Paranitrobiphenyl, a new bladder carcinogen in the dog. *Ind. Med. Surg.*, **27**, 634.
102. WOGAN, G. N., PAGLIALUNGA, S., ARCHER, M. C. & TANNENBAUM, S. R. (1975) Carcinogenicity of Ephedrine, Sarcosine, Folic acid and Creatinine. *Cancer Res.*, **35**, 1981.
103. KAWAZOE, Y., TACHIBANA, M., AOKI, K. & NAKAHARA, W. (1969) The structure carcinogenicity relationship among derivatives of 4-nitro and 4-hydroxylaminoquinoline 1-oxides. *Biochem. Pharmacol.*, **16**, 631.
104. BARNE, H. G., YEE, H. T. & SEFERIAN, S. (1968) The toxicity of rubber additives. Findings from a survey of 140 plants in Ohio. *Archs. Environ. Health*, **16**, 700.
105. ROGERS, S. (1957) Inhibitory influence of a normally occurring pyrimidine precursor upon methylcholanthrene carcinogenesis. *Proc. Soc. exp. Biol.*, **96**, 464.
106. FLETCHER, K. (1972) ICI Report HO/IH/P/21.
107. FLETCHER, K. (1972) ICI Report IHR 185.
108. FINZI, C., DAUDEL, P. & PRODI, G. (1968) Interference among polycyclic hydrocarbons in experimental skin carcinogenesis. *Eur. J. Cancer*, **3**, 497.
109. ULLAND, B., FINKELSTEIN, M., WEISBURGER, E. K., RICE, J. M. & WEISBURGER, J. H. (1971) Carcinogenicity of industrial chemicals propylene imine and propane sultone. *Nature*, **230**, 460.
110. PARISH, D. J. & SEARLE, C. E. (1966) The carcinogenicity of β-propiolactone and 4-nitroquinoline-N-oxide for the skin of the golden hamster. *Br. J. Cancer*, **20**, 200.
111. MINER, D. L., MILLER, J. A., BARMAN, C. A. & RUSCH, H. P. (1943) The effect of pyridoxin and other B vitamins on the production of liver cancer with p-dimethylaminoazobenzene *Cancer Res.*, **3**, 296.
112. LONG, E. L., NELSON, A. A., FITZHUGH, O. G. & HANSEN, W. H. (1963) Liver tumours produced in rats by feeding safrole. *Archs. Path.*, **75**, 595.
113. HOLLAND, V. R., SAUNDERS, B. C., ROSE, F. L. & WALPOLE, A. L. (1974) A safer substitute for benzidine in the detection of blood. *Tetrahedron*, **30**, 3299.
114. SPITZ, S., MAGUIGAN, W. H. & DOBRINGER, K. (1950) The carcinogenic action of benzidine. *Cancer*, **3**, 789.
115. FREI, J. V. & KINGSLEY, W. F. (1968) Observations on chemically induced regressing tumours

TABLE II.—*Numbers of Compounds Correctly Identified by Short-term Tests*

| Class of compound | Total number tested | Number of compounds identified correctly: Bacterial mutation | Cell transformation | Degranulation | Sebaceous-gland suppression | Tetrazolium reduction | Implant |
|---|---|---|---|---|---|---|---|
| Polycyclic | 20 | 19 | 19 | 13 | 18 | 10 | 15 (17) |
| Carcinogens | 11 | 11 | 10 | 8 | 11 | 5 | 6 (8) |
| Non-carcinogens | 9 | 8 | 9 | 5 | 7 | 5 | 9 |
| Arylamine | 33 | 31 | 32 | 25 | 20 (32) | 18 (32) | 18 (30) |
| Carcinogens | 20 | 19 | 19 | 19 | 11 | 8 | 7 (18) |
| Non-carcinogens | 13 | 12 | 13 | 6 | 9 (12) | 10 (12) | 11 (12) |
| Alkylating agent | 18 | 15 | 17 | 11 | 12 | 7 | 4 (16) |
| Carcinogens | 18 | 15 | 17 | 11 | 12 | 7 | 4 (16) |
| Non-carcinogens | 0 | — | — | — | — | — | — |
| Miscellaneous | 49 | 46 | 45 | 36 | 28 | 32 (48) | 38 (47) |
| Carcinogens | 9 | 8 | 7 | 3 | 5 | 3 | 2 |
| Non-carcinogens | 40 | 38 | 38 | 33 | 23 | 29 (39) | 36 (38) |
| Total of all classes | 120 | 111 | 113 | 85 | 78 (119) | 67 (118) | 75 (110) |
| Carcinogens | 58 | 53 | 53 | 41 | 39 | 23 | 19 (51) |
| Non-carcinogens | 62 | 58 | 60 | 44 | 39 (61) | 44 (60) | 56 (59) |

In parentheses, the total numbers tested, when different from numbers in Column 2.

of mouse epidermis. *J. natn. Cancer Inst.*, **41**, 1307.

116. Epstein, S. S., Arnold, E., Andrea, J., Bass, W. & Bishop, Y. (1972) Detection of chemical mutagens by the dominant lethal assay in the mouse. *Toxicol. appl. Pharmacol.*, **23**, 288.
117. Deringer, M. K. (1962) Response of strain HR/De mice to painting with urethane. *J. natn. Cancer Inst.*, **29**, 1107.
118. Jaffe, W. G. (1947) Carcinogenic action of ethyl urethane on rats *Cancer Tes.*, **7**, 107.
119. Mohr, U., Reznik, G. & Reznik-Schuller, H. (1974) Urethane as a carcinogen for the European Hamster. *J. natn. Cancer Inst.*, **53**, 1359.
120. Maltoni, C. & Lefemine, G. (1974) Carcinogenicity bioassays on vinyl chloride 1. Research plan and early results. *Environ. Res.*, **7**, 387.
121. Viola, P. L., Bigotti, A. & Caputo, A. (1971) Oncogenic response of rat skin, lung and bones to vinyl chloride. *Cancer Res.*, **31**, 51.6

Fig. 1.—The effect of variations in p, the the prior probability that a compound is a carcinogen (or % of carcinogen in the samples being tested) on P the probability that a compound producing a particular test result is a carcinogen. Each curve represents a particular test result: (a) bacterial mutation and cell transformation positive; (b) cell transformation positive; (c) bacterial mutation positive; (d) bacterial mutation negative and cell transformation positive; (e) bacterial mutation positive and cell transformation negative; (f) bacterial mutation negative; (g) cell transformation negative; (h) bacterial mutation and cell transformation negative. The curves are calculated from the formula:

$$P = \frac{pA}{pA + (1 - p)B}$$

A is the probability of obtaining the test result with carcinogens, and B is the probability of obtaining the test result with non-carcinogens. The values for A and B were obtained from this study.

### DISCUSSION

The 6 tests compared in this paper were developed in several laboratories and each has been previously validated to different extents. The test which has been most extensively used is that developed by Ames and his colleagues, and results from testing over 300 chemicals have recently been reported (McCann et *al.*, 1975). This is the first comparative blind study of several tests carried out in one laboratory.

The results of a validation study of this type will be affected by a variety of factors, which include the choice and classification of chemicals, the inherent reproducibility of the test systems, and the fact that these experiments were not repeated.

The chemicals used in this study were selected to represent a wide range of

Fig. 2.—The effect of variations in p the prior probability that a compound is a carcinogen (% of carcinogens in the samples being tested) on P, the probability that a compound producing a particular test result is a carcinogen. Curves d and dd for the test result bacterial mutation negative and cell transformation positive; e and ee for bacterial mutation positive and cell transformation negative. Curves d and e as in Fig. 1. Curves dd and ee are the false-positive results, calculated from the formula

$$P = \frac{(1 - p)B}{pA + (1 - p)B}$$

(symbols as in Fig. 1).

structures, and included many of the organic chemicals which are carcinogens in animals and most of those which are known to be active in man, but inevitably some classes of carcinogen are not represented. The effect of this selection on the results of the validation study are difficult to estimate, and care should be taken when extrapolating these predictivity figures to chemicals of a new structural type, as discussed later. The classification of the chemicals as carcinogenic or

FIG. 3a.—Mutagenic response of *Salmonella typhimurium* strains TA 1535, TA 1538, TA 100 and TA 98 to carcinogenic and non-carcinogenic pairs of structurally related compounds. ●——● Dimethylcarbamoyl chloride; ○- - - -○ Dimethylformamide.

FIG. 3b.—●——● 2,4-Dinitrofluorobenzene; ○- - - -○ 1,3-Dinitrobenzene.

non-carcinogenic was, in most cases, relatively easy. Nevertheless, some of the criteria used are controversial. Thus, there may be disagreement with the classification of DDT, phenobarbital and dieldrin, which only produce an increase in hepatomas or pulmonary adenomas in mice, as non-carcinogens. Other compounds which presented difficulties in classification are 1-fluoro-2,4-dinitrobenzene, croton oil and 3,3-diaminobenzidine. Since there were few compounds within this category the effect of any changes on the overall results would be relatively small.

*Accuracy of the tests*

A comparison of the performance of

FIG. 3c.—●——● 2-Naphthylamene; ○- - - -○ 1-Naphthylamine.

the tests for carcinogens and non-carcinogens in each chemical class is given in Tables II and III. The percentages have been calculated on the number of compounds actually tested; but, because the chemical classes had differing proportions of carcinogens and non-carcinogens, the data have been transformed for equal numbers of carcinogens and non-carcinogens in each class for comparative purposes.

Table VII is an abstract of the results in Table I for structurally related carcinogen and non-carcinogen pairs, first reported by Purchase *et al.* (1976). The Ames test correctly distinguished the 8 pairs of compounds, whereas cell transformation failed to identify 2-naphthyl-

FIG. 3d.—●——● Nitrosofolic acid; ○- - - -○ Diphenylnitrosamine.

amine as a carcinogen. The dose-response curves obtained for the pairs of compounds using the 2 tests are shown in Figs. 3–6. It can be seen that in the cell-transformation test of 2-naphthylamine the transformation frequency rose rapidly at doses greater than the $LC_{50}$. Subsequent testing gave a positive result with 2-naphthylamine suggesting that the failure of the test reported here was for technical reasons.

Short-term tests will usually be conducted on compounds with unknown carcinogenic activity. It is important that the tests should be accurate for detecting both carcinogens and non-carcinogens as well as having a high overall accuracy. A tabulation of the percentage of positive and negative results for both carcinogens and non-carcinogens for each test is given in Table IV. The differences between the percentage of positive results for carcinogens and non-carcinogens is statistically significant ($\chi^2$, $P < 0{\cdot}05$) for all tests except the tetrazolium test, indicating that 5 of the tests had *some* ability to discriminate between carcinogens and non-carcinogens. The ratio of positive results for carcinogens and non-carcinogens is a measure of the discriminating power. This ratio, and the ratio for negative results, are given in Table IV. It is

FIG. 4a.—Mutagenic response of *Salmonella typhimurium* strains TA 1535, TA 1538, TA 100 and TA 98 to carcinogenic and non-carcinogenic pairs of structurally related compounds. ●——● 4-Nitroquinoline-N-oxide; ○- - - -○ 3-Methyl-4-nitroquinoline-N-oxide.

FIG. 4b.—●——● Benzidine; ○- - - -○ 3,3′,5,5′-Tetramethylbenzidine.

only the cell transformation and bacterial-mutation tests which combine a high predictive accuracy with a low level of false results (indicated by the high ratios in Table IV). Although the observed levels of accuracy varied for each test between the 4 classes of compounds, there was statistical evidence of real differences in accuracy for only one of the tests ($\chi^2$, $P < 0{\cdot}05$): the degranulation test. (Predictive accuracy for carcinogens varied from 95% to 33% and for non-carcinogens 82% to 46%.)

*Independence of test results*

If the tests were not independent, *i.e.*

FIG. 4c.—●——● 2-Acetylaminofluorene; ○- - - -○ 4-Acetylaminofluorene.

they all had the same positive, negative and false results, the use of more than one test would be unhelpful, because each test used in addition to the first one would merely be duplicating the results. The 15 possible pairs from the 6 tests have been considered for evidence of non-independence, separately for carcinogens and non-carcinogens. There was no such evidence ($\chi^2$ test) for non-carcinogens for any pair of tests. For carcinogens, however, there was statistical evidence of non-independence for the tetrazolium and implant results. For these 2 tests the proportion of compounds recorded as negative by the first test was higher for those negative in the second test than for those positive in the second test. This general low level of

FIG. 4d.—●——● 9,10-Dimethylanthracene; ○- - - -○ Anthracene.

dependence suggests that it might be worthwhile using a battery of tests for unknown compounds.

*A test battery*

The idea of using a battery of tests appears at first sight to provide considerable advantages, particularly if the objective is to increase the probability of detecting all carcinogens. In using results from more than one test, the interpretation is relatively easy if all tests agree.

FIG. 5a.—Survival curves in liquid culture and frequency of transformation as assessed by colony formation in semi-solid agar of BHK 21 cells after dosing with carcinogenic and non-carcinogenic pairs of structurally related compounds. ●——● 4NQO; ▲---▲ 3Me4NQO.

FIG. 5c.—●—● 9,10-Dimethylanthracene; ▲---▲ Anthracene.

FIG. 5b.—●——● Benzidine; ▲---▲ Tetramethylbenzidine.

FIG. 5d.—●——● 2AAF; ▲---▲ 4AAF.

FIG. 6a.—Survival curves in liquid culture and frequency of transformation as assessed by colony formation in semi-solid agar of BHK 21 cells after dosing with carcinogenic and non-carcinogenic pairs of structurally related compounds. ●——● Dimethylcarbamoyl chloride; ▲- - -▲ Dimethylformamide.

FIG. 6c.—●——● 2-Naphthylamine; ▲- - -▲ 1-Naphthylamine.

FIG. 6b.—●——● Dinitrofluorobenzene; ▲- - -▲ Dinitrobenzene.

FIG. 6d.—●——● Nitrosofolic acid; ▲- - -▲ Diphenylnitrosamine.

TABLE III.—*Percentage Accurate Predictions of Short-term Tests*

| Class of compound | % Accurate predictions: Bacterial mutation | Cell trans-formation | Degranula-tion | Sebaceous-gland suppression | Tetrazolium reduction | Implant |
|---|---|---|---|---|---|---|
| Polycyclic | 95 | 95 | 65 | 90 | 50 | 88 |
| Carcinogens | 100 | 91 | 73 | 100 | 45 | 75 |
| Non-carcinogens | 89 | 100 | 55 | 78 | 55 | 100 |
| Arylamine | 94 | 97 | 76 | 62 | 56 | 60 |
| Carcinogens | 95 | 95 | 95 | 55 | 40 | 39 |
| Non-carcinogens | 92 | 100 | 46 | 75 | 83 | 92 |
| Alkylating agent | 83 | 94 | 61 | 67 | 39 | 25 |
| Carcinogens | 83 | 94 | 61 | 67 | 39 | 25 |
| Non-carcinogens | — | — | — | — | — | — |
| Miscellaneous | 94 | 92 | 73 | 57 | 67 | 81 |
| Carcinogens | 89 | 78 | 33 | 55 | 33 | 22 |
| Non-carcinogens | 95 | 95 | 82 | 57 | 74 | 95 |
| Total of all classes | 93 (92) | 94 (93) | 71 (64) | 65 (70) | 57 (51) | 68 (59) |
| Carcinogens | 91 (96) | 91 (90) | 71 (65) | 67 (69) | 40 (40) | 37 (23) |
| Non-carcinogens | 94 (96) | 97 (97) | 71 (62) | 64 (71) | 73 (71) | 95 (95) |

In parentheses, corrected for equal numbers of carcinogens and non-carcinogens in each chemical class.

TABLE IV.—*Percentage of Positive or Negative Results Obtained with Each Test for Both Carcinogens and Non-carcinogens*

| Test | Positive results (%) | | | Negative results (%) | | |
|---|---|---|---|---|---|---|
| | Carcinogens | Non-carcinogens | Ratio | Carcinogens | Non-carcinogens | Ratio |
| Cell transformation | 91 | 3 | 30 | 9 | 97 | 11 |
| Bacterial mutation | 91 | 6 | 15 | 9 | 94 | 10 |
| Implant | 37 | 5 | 7·4 | 63 | 95 | 1·5 |
| Degranulation | 71 | 29 | 2·4 | 29 | 71 | 2·4 |
| Sebaceous gland | 67 | 36 | 1·9 | 33 | 66 | 2·0 |
| Tetrazolium | 40 | 27 | 1·5 | 60 | 73 | 1·2 |

TABLE V.—*Performance of Short-term Tests, Alone or in Combinations*

| Tests | % of carcinogens which would be +ve in at least one test | % of non-carcinogens which would be +ve in at least one test |
|---|---|---|
| CT | 91 | 3 |
| CM | 91 | 6 |
| Deg | 71 | 29 |
| SI | 37 | 5 |
| SG | 67 | 36 |
| TR | 40 | 27 |
| CT+BM | 99·19 | 8·8 |
| CT+BM+Deg | 99·77 | 35·3 |
| CT+BM+SI | 99·49 | 13·4 |
| CT+BM+Deg+SI | 99·85 | 38·5 |
| CT+BM+Deg+SG | 99·92 | 58·6 |
| CT+BM+SI+SG | 99·83 | 44·6 |
| CT+BM+Deg+SI+SG | 99·95 | 60·6 |
| CT+BM+Deg+SG+TR | 99·95 | 69·8 |
| CT+BM+SI+TR | 99·90 | 59·5 |
| All 6 tests | 99·97 | 71·33 |

* CT = cell transformation, BM = bacterial mutation, Deg = degranulation. SI = subcutaneous implants, SG = sebaceous gland, TR = tetrazolium reduction.

*NB:*

1. Based on the results obtained in this study and assumptions stated in the discussion.
2. The number of decimal places does not represent the likely accuracy of these figures but demonstrates the likely size of the differences between the examples given.

TABLE VI.—*Compounds which Produced Different or False Results in the Cell Transformation and Bacterial Mutation Tests*

| | Bacterial mutation | Cell transformation | Animal carcinogenicity |
|---|---|---|---|
| 3,3′-Diaminobenzidine | + | − | − |
| 1-Naphthol | + | − | − |
| Perylene | + | − | − |
| Trimethylphosphate | + | − | − |
| 2-Naphthylamine | + | − | + |
| Diethylstilboestrol | − | − | + |
| Vinyl chloride | − | − | + |
| 6-Benzoyl-2-naphthol | − | + | − |
| Hydrocortisone | − | + | − |
| Croton oil | + | − | + |
| 2,7-Diaminofluorene | − | + | + |
| 3,4,5,6-Dibenzacridine | + | − | + |
| Dimethylnitrosamine | − | + | + |
| DL-Ethionine | − | + | + |

TABLE VII.—*Response of the 6 Short-term Tests to 8 Carcinogen/Non-carcinogen Pairs*

| Test compound | Ames test | Cell transformation | Rabin's test | Subcutaneous implants | Sebaceous-gland suppression | Tetrazolium reduction | Animal carcinogenicity |
|---|---|---|---|---|---|---|---|
| 4-Nitroquinoline-N-oxide | + | + | + | + | + | + | + |
| 3-Methyl-4-nitroquinoline-N-oxide | − | − | − | − | − | − | − |
| Benzidine | + | + | + | − | + | − | + |
| 3,3′,5,5′-Tetramethylbenzidine | − | − | + | − | − | − | − |
| 2-Acetylaminofluorene | + | + | + | − | − | − | + |
| 4-Acetylaminofluorene | − | − | + | * | * | * | − |
| 9,10-Dimethylanthracene | + | + | − | + | + | + | + |
| Anthracene | − | − | + | − | − | − | − |
| Dimethylcarbamoyl chloride | + | + | − | + | + | − | + |
| Dimethylformamide | − | − | − | − | − | − | − |
| 1-Fluoro-2,4-dinitrobenzene | + | + | − | + | + | − | + |
| 1,3-Dinitrobenzene | − | − | − | − | + | − | − |
| 2-Naphthylamine | + | − | + | − | + | + | + |
| 1-Naphthylamine | − | − | + | − | − | − | − |
| Nitrosofolic acid | + | + | + | − | − | − | + |
| Diphenylnitrosamine | − | − | − | − | + | + | − |
| Number of pairs correctly identified | 8 | 7 | 2 | 4 | 5 | 3 | |

* Not tested.

In some cases, however, the tests will not all agree (see Table VI). When the tests disagree, and no information is available about the relative performance of the test with that chemical class, it is likely that a positive result in any test will be considered to indicate that the compound is a carcinogen. This process is likely to increase the number of false-positive results. The performance of the 6 tests used for this study in various combinations is given in Table V. As one would expect, the ability to detect carcinogens (the percentage of carcinogens giving a positive result in at least one test) increases as additional tests are used, but the advantage obtained is not great when tests in addition to cell transformation and bacterial mutation are used. The number of false positives (the percentage non-carcinogens which are positive in at least one test) also increases as the number of tests increases. Using cell transformation and bacterial mutation, the 2 "best" tests, the false positive results increase from 3% or 6% respectively to 9%. When all 6 tests are used, 44/62 non-carcinogens (71%) were positive in at least one test. A balance is clearly necessary between the ability of the tests to detect all carcinogens and the proportion of false positives produced. On the basis of the figures given in Table V, the combination of cell transformation and bacterial mutation gives some advantage over the use of one test alone.

*The use of the 2 'best' tests*

It is noteworthy that the combination of the cell transformation and bacterial mutation tests on the 120 compounds detected all but 2 of 58 carcinogens, and gave a positive result for only 6 of the 62 non-carcinogens. The relative importance of these "false" positive and "false" negative results when testing groups of compounds will vary according to the ratio of carcinogens to non-carcinogens in the group of compounds being tested. This effect, based on a hypothetical test that was 90% accurate for both carcinogens and non-carcinogens, was described in our preliminary report (Purchase *et al.*, 1976). Using the results obtained in this study, and again making a number of assumptions which are stated later in this discussion, Fig. 1 and 2 have been obtained. Fig. 2 demonstrates the effect of varying the proportion of carcinogens in the compounds tested on the probability that a compound which is positive in the bacterial mutation or cell transformation test is a carcinogen. Two lines are drawn in Fig. 1; one for the proportion of carcinogens in the compounds judged as positive and the other for the proportion of non-carcinogens in these compounds where the cell transformation is positive and the bacterial mutation is negative. (The 2 proportions must obviously sum to one.) The lines for the alternative result, namely cell transformation negative and bacterial mutation positive, are very similar and have not been presented in Fig. 2.

It can be seen from Fig. 1 that the use of a combination of the 2 tests results in a more confident prediction of the carcinogenicity of the compound when they agree than if a single test had been used. However, if the 2 tests disagree, little further information is gained about the potential carcinogenicity of the compound.

If, for a given group of compounds to be tested, an estimate of the proportion of carcinogens is available, the proportion of carcinogens with a given result can be read from figures like Fig. 1. Alternatively, study of the chemical structure of a compound may enable us to make an estimate of the probability of it being a carcinogen, which is equivalent to estimating the proportion of carcinogens in that class of compounds.

The calculations on which the graphs (Fig. 1 and 2) are based make certain assumptions. These are: (a) that the 120 compounds used in this study are representative of the compounds to be tested; (b) that the results of the cell-transformation and bacterial-mutation tests are independent; (c) that the tests have the same degree of reproducibility in this study as they will when used in future and (d) that the classification of the 120 compounds into carcinogens and non-carcinogens is correct.

In a future situation, a number of these assumptions may not be valid; *e.g.* it is unlikely that the 120 compounds used are representative of all groups of compounds to be tested in future and recent evidence (Huberman *et al.*, 1976) suggests that cell transformation occurs as a result of a mutational event which may be similar to the reverse mutation in Salmonella. It would be advantageous to gather information about the carcinogenicity and short-term results of structurally related compounds when testing a new compound; to do so would alter the first assumption above in favour of a more accurate result. This theme is developed further in Appendix I. The preceding discussion considers the application of tests to large numbers of compounds, and the consequences in terms of accuracy of prediction. When these tests are used in practice, however, results will have to be assessed on a single compound or a group of compounds within a chemical class. A different set of considerations then has to be taken into account.

*The practical use of short-term tests*

Short-term tests do not have cancer induction as their end-point; rather they each have some parameter which will vary with the carcinogenicity or non-carcinogenicity of the test compound. Those already using, or advocating the use of short-term tests to detect potential chemical carcinogens should be aware of the constraints under which such tests operate.

The *first* constraint is that the test parameters should not be given a status greater than that of an arbitrary response, irrespective of how biologically significant any one test might appear to be with respect to the theories of the chemical induction of cancer (Boveri, 1914; Bauer, 1928; Burdette, 1955; Brookes and Lawley, 1964; Miller and Miller, 1971*a*, *b*; Ames *et al.*, 1973). The *second* constraint is that, however many generalized data might be generated to support the predictive accuracy of a given test (McCann *et al.*, 1975; Purchase *et al.*, 1976; Bartsch *et al.*, 1976) this accuracy should not be assumed to apply uniformly to compounds of every chemical class. Therefore, any test should be assessed by the correlation between the results from this test and the known *in vivo* carcinogenicity within the class of compound being studied. It follows that a single test may not be sufficient to cover all classes of compounds, and it may be necessary to evaluate potential carcinogenicity within a given group of compounds with a test other than that which is generally used. The accuracy of a short-term test for carcinogenicity has been defined in this study as the percentage of carcinogens which are positive in the test. The Ames and cell-transformation tests are able to identify more than 90% of carcinogens, and on the basis of this and other work the Ames test is the most satisfactory established test. However, relatively little attention has been given to the converse problem of how many compounds shown to be positive in a short-term test are, in fact, carcinogens. On the basis of the high-predictivity figures mentioned above, one would expect a high proportion of compounds shown to be positive in the Ames or cell transformation tests to be carcinogens. If this is so, the tests will be extremely useful in predicting carcinogenicity, irrespective of any other biological significance of the positive result. Nevertheless, attention should be given to the biological significance, apart from carcinogenicity, of a positive result in a test. This is particularly so if the percentage of carcinogenic compounds shown to be positive in the test is relatively low. A positive result in the Ames test indicates that a chemical has induced genetic change, presumably by interaction with DNA. Irrespective of how well the Ames test results correlate with carcinogenicity, the potential mutagenicity of a compound shown to be positive must be considered, and any correlation between positive results in the Ames test and mammalian mutagenicity will need to be established independently. The significance of a positive result in the cell-transformation assay in terms of biological phenomena other than carcinogenicity is not so obvious, although cell transformation too may be a mutational event (Huberman *et al.*, 1976). The suitability of cell transformation as an indicator of mammalian mutagenicity will also need to be established. With the sebaceous-gland, degranulation, subcutaneous-implant and tetrazolium-reduction tests the significance of a positive result in terms other than carcinogenicity is not immediately apparent.

Although the previously published data for the Ames test and the data generated by this study for both the Ames and cell-transformation tests, indicate that both

TABLE VIII.

| Compound | Ames test | Cell transformation | Animal carcinogenicity |
|---|---|---|---|
|  (I) | + | + | + |
|  | + | + | + |
|  | + | + | + |
|  | + | + | + |
|  (II) | – | – | – |

are reliable and highly predictive carcinogenicity tests, the following examples illustrate the difficulties which can be met with when these or similar tests are used for routine screening.

Table VIII shows the results obtained in this study using these 2 tests with derivatives of benzidine. The results indicate that both test systems are able to identify benzidine (I) and its carcinogenic analogues as mutagens or transforming agents, whilst finding a structurally related, but non-carcinogenic analogue such as 3,3′5,5′-tetramethylbenzidine (II) negative.

This test-response profile means that confidence can be placed in the findings of either of these test systems for previously unevaluated derivatives of benzidine. Furthermore, when testing such derivatives with either of these tests, their continuing ability to discriminate between carcinogens and non-carcinogens of this class can be monitored by using benzidine and 3,3′5,5′-tetramethylbenzidine as the *chemical class control pair* (CCCP). This practice would rapidly reveal any critical changes in the test system, which may have unknowingly occurred since the initial exploratory study was carried out. For example, it has been demonstrated (Oesch *et al.*, 1976) that a test response can be critically manipulated by variations in a single enzyme in the S9 mix. The concept that a test should be capable of correctly identifying the appropriate CCCP before analogues are evaluated is a useful method of discerning false results. For example, hydrazine (III), hexamethylphosphoramide (IV), safrole (V), urethane (VI) and diethylstilboestrol (DES) (VII) have been associated with

$NH_2—NH_2$
(III)

$[(CH_3)_2N]_3PO$
(IV)

(V)

$H_2N—CO_2Et$
(VI)

(VII)

induction of tumours in rodents. Furthermore, each of these compounds can be associated with a number of structural analogues, any one of which might also be carcinogenic and would, therefore, be worthy of evaluation in an *in vitro* test. However, as there is no negative compound to form the CCCP before these structural analogues are tested, it is important to first study the response of the chosen test to the reference carcinogen. In the examples cited above each has been tested in the Ames test and each gives a negative (as in the case of DES) or erratic response. Therefore, in any situation where an erratic response is obtained the ability of the test to identify the reference carcinogen should be demonstrated in each experiment. Only under such circumstances can negative results be used to dissociate analogues from the *in vivo* carcinogenicity of the parent carcinogen. There is, consequently, a hidden danger in the practice of establishing a short-term test and only checking its "sensitivity" with chemically unrelated carcinogens. For example a positive response given by 2AAF, although acting as a test system control, would not automatically guard against potentially carcinogenic analogues of safrole from passing undetected.

One solution to this problem would be for those engaged in the routine testing of chemicals to gather together a collection of carcinogenic and non-carcinogenic analogues from as many discrete classes of chemical carcinogens as possible to act as chemical-class controls. It should then be possible to select in advance the most appropriate short-term test with which to evaluate a structurally coherent series of compounds simply by testing the appropriate controls in a variety of tests and choosing the test with the best response in Table II.

For example, the CCCP formed by nitrosofolic acid and diphenylnitrosamine was correctly identified only by the Ames, cell-transformation and degranulation tests (Table IX). The implant test appears to detect nitrosamines as negative, irrespective of their carcinogenic or non-carcinogenic properties. The sebaceous gland test and the tetrazolium-reduction tests have

TABLE IX.

| Test compound | Ames' test | Cell transformation | Rabin's test | Subcutaneous implants | Sebaceous-gland suppression | Tetrazolium reduction | Animal carcinogenicity |
|---|---|---|---|---|---|---|---|
| Nitrosofolic acid | − | + | + | − | − | − | + |
| Diphenylnitrosamine | − | − | − | − | + | + | − |
| HMPA | ± | + | − | − | − | − | + |
| Diethylstilboestrol | − | − | − | − | − | + | +? |

a response inverse to carcinogenicity. These latter 3 tests are consequently unsuitable for the evaluation of the potential carcinogenicity of nitrosamines, and it would clearly be wrong to draw conclusions from any positive or negative results given by these 3 tests for a nitrosamine. In addition, given that the first 3 tests are suitable for the evaluation of the potential carcinogenicity of nitrosamines, it would be better to choose either of the first 2 tests rather than the degranulation test, on the basis of the overall reliability gradings of these tests. Similarly, since the response of the Ames test to the carcinogen HMPA (IV) is erratic, the cell-transformation test has been defined in advance as the better test with which to evaluate analogues of HMPA. None of the 6 tests is suitable to evaluate analogues of DES. It is currently possible to define and synthesize about 20 CCCPs from the various classes of carcinogens. These pairs, together with a variety of known animal carcinogens which as yet have no well defined non-carcinogenic analogues (such as hydrazine and aflatoxin $B_1$) could be used to select and monitor the most responsive test for a particular class of test compounds and also to critically compare new or developing short-term test systems.

It is not always possible to select appropriate chemical-class controls. When this situation occurs it should either be clearly accepted (especially if negative results occur) or an attempt should be made to establish *ab initio* a standard carcinogen and non-carcinogen for the new class by means of conventional long-term animal studies.

### "FALSE" RESULTS

### *False negatives*

Negative short-term predictions for an established animal carcinogen, or a compound ultimately capable of being shown to be such, could be anticipated to occur for 2 main reasons. The first may be because the carcinogenicity of the compound was not assessed *via* a sensitive test. Thus, carcinogens which elicit their effect by a disturbance of a hormonal mechanism (such as diethylstilboestrol) or *via* a solid-state mechanism (such as asbestos or plastic implants) would not necessarily be expected to give positive results in short-term tests. Likewise, a lack of response by such tests could be anticipated for purely inorganic carcinogens where *direct* covalent interaction with DNA is unlikely to occur, and for compounds whose carcinogenicity results from continual physico-chemically induced tissue damage and its resultant repair (for example repeated s.c. injections of hypertonic solutions or repeated liver damage from some hepatotoxins). It is also not yet clear whether chemicals which are thought to produce cancer *via* free-radical formation will be detected by those tests. The second area of anticipated failure has been discussed above and elsewhere (Ashby *et al.*, 1977) and concerns the selection and optimization of the best test for a particular class of potential carcinogens.

### *False positives*

Although positive results generated for animal non-carcinogens appear to offer a smaller problem, their widespread occurrence would make them significant. Again, there are 2 major potential causes of such results. The first is that the animal study is inadequate. For example, a non-sensitive species may have been selected for testing or the route of administration of the compound failed to maximize its carcinogenic potential. Alternatively the study may have been terminated too soon or the pathology of the animals was inadequate. The latter 2 objections apply particularly to most of the currently known "false-positive" results, as they are often based upon older animal studies, conducted with protocols which would be unacceptable by today's standards. There will, however, remain a nucleus of *genuine* false-positive predictions due to the gross simplicity of any test when compared to whole animal absorption, distribution, metabolism (both detoxification and carcinogenic activation) and excretion of compounds. Further such tests do not allow for the normal protective mechanisms which operate *in vivo*, such as DNA repair, immunological surveillance and death prior to overt cancer induction. In particular, the selection of dose levels for an animal study may be critical to the outcome of the experiment, depending upon whether or not such protective mechanisms are maintained intact during the study.

Finally it is too early to discount the possibility that an otherwise reliable test may respond positively to chemicals of a particular type due to specific factors which are not associated with carcinogenicity.

The Authors would like to thank Mr. T. Weight for the statistical analysis, Mrs Lynn Henry, Miss Judith Naden, Mrs Susan Schofield and Mr M. Scholes for technical assistance, and Mrs Beryl Syrotiuk for typing.

### REFERENCES

AMES, B. N., DURSTON, W. E., YAMASAKI, E. & LEE, F. D. (1973) Carcinogens are Mutagens: a Single Test System Combining Liver Homogenates for Activation and Bacteria for Detection. *Proc. natn. Acad. Sci. U.S.A.*, **70**, 2281.

AMES, B. N., MCCANN, J. & YAMASAKI, E. (1975) Methods for Detecting Carcinogens and Mutagens with the Salmonella/Mammalian-microsome Mutagenicity Test. *Mutation Res.*, **31**, 347.

ARCOS, J. C. & ARGUS, M. F. (1974) *Chemical Induction of Cancer.* New York: Academic Press.

ASHBY, J., STYLES, J. A. & ANDERSON, D. (1977) Selection of an *In vitro* Carcinogenicity Test for Derivatives of the Carcinogen Hexamethylphosphoramide. *Br. J. Cancer*, **36**, 564.

BARTSCH, H., MALAVEILLE, C. & MONTESANO, R. (1976) The Predictive Value of Tissue-mediated Mutagenicity Assays to Assess to Carcinogenic Risk of Chemicals. In: *IARC Scientific Publication No.* 12. *Screening Tests in Chemical Carcinogenesis.* p. 467.

BAUER, K. H. (1928) Mutations Theorie der Geschwulst-Enstehung Ubergang von Korperzellen In *Geschwulstzellen durch Gen-Anderung.* Berlin: Springer.

BOCK, F. H. & MUND, R. (1958) A Survey of Compounds for Activity in the Suppression of Mouse Sebaceous Glands. *Cancer Res.*, **18**, 887.

BOVERI, T. (1914) *Zur Frage der Entstehung maligner Tumoren.* Jena: Gustav Fischer.

BOYLAND, E. (1969) Correlation of Experimental Carcinogenesis and Cancer in Man. *Prog. exp. Tumour Res.*, **11**, 222.

BRIDGES, B. A. (1976) Short Term Screening Tests for Carcinogens. *Nature*, **261**, 195.

BROOKES, P. (1971) On the Interaction of Carcinogens with DNA. *Biochem. Pharmacol.*, **20**, 999.

BROOKES, P. & LAWLEY, P. D. (1964) Evidence for the Binding of Polynuclear Aromatic Hydrocarbons to the Nucleic Acids of Mouse Skin: Relation between Carcinogenic Power of Hydrocarbons and their Binding to Deoxyribonucleic Acid. *Nature*, **202**, 781.

BROOKES, P. & DE SERRES, F. (1976) Report on the Workshop on the Mutagenicity of Chemical Carcinogens, Honolulu, 1974. *Mutation Res.*, **88**, 155.

BURDETTE, W. J. (1955) The Significance of Mutation in Relation to the Origin of Tumours: a Review. *Cancer Res.*, **15**, 201.

CAIRNS, J. (1975) The Cancer Problem. *Sci. Am.*, **233**, 64.

CLAYSON, D. B. (1962) *Chemical Carcinogenesis.* London: Churchill.

DINMAN, B. D. (1974) *The Nature of Occupational Cancer.* Springfield: Charles C. Thomas.

HIGGINSON, J. (1969) Present Trends in Cancer Epidemiology. In *Canadian Cancer Conf.* Ed. J. F. Morgan. Oxford: Pergamon Press. p. 40.

HIGGINSON, J. & MUIR, C. S. (1973) Epidemiology. In *Cancer Medicine.* Eds. J. F. Holland and E. Frei, III. Philadelphia: Lea and Febiger. p. 241.

HUBERMAN, E., MAGER, R. & SACHS, L. (1976) Mutagenesis and Transformation of Normal Cells by Chemical Carcinogens. *Nature*, **264**, 360.

HUEPER, W. C. & CONWAY, W. D. (1964) *Chemical Carcinogenesis and Cancers.* Springfield: Charles C. Thomas.

IVERSEN, O. H. & EVENSEN, A. (1962) *Experimental Skin Carcinogenesis in Mice.* Norwegian Univ. Press.

MACPHERSON, I. & MONTAGNIER, L. (1964) Agar Suspension Culture for the Selective Assay of Cells Transformed by Polyoma Virus. *Virology*, **23**, 291.

MCCANN, J., CHOI, E., YAMASAKI, E. & AMES, B. N. (1975) Detection of Carcinogens as Mutagens in the Salmonella/Microsome Test. Part I, Assay of 300 Chemicals. *Proc. natn. Acad. Sci. U.S.A.*, **72**, 5135.

MILLER, J. A. (1970) Carcinogenesis by Chemicals: an Overview. *Cancer Res.*, **30**, 559.

MILLER, J. A. & MILLER, E. C. (1971*a*) Chemical Carcinogenesis: Mechanisms and Approaches to its Control. *J. natn. Cancer Inst.*, **47**, 5.

MILLER, E. C. & MILLER, J. A. (1971*b*) The Mutagenicity of Chemical Carcinogens: Correlations, Problems and Interpretations. In *Chemical Mutagens*, Vol. 1. Ed. A. Hollaender. New York: Plenum Press. p. 83.

MILLER, E. C. & MILLER, J. A. (1972) Approaches to the Mechanisms and Control of Chemical Carcinogenesis. In *Environment and Cancer.* Baltimore: Williams and Wilkins. p. 5.

MILLER, J. A. & MILLER, E. C. (1974) Some Current Thresholds of Research in Chemical Carcinogenesis. In *Chemical Carcinogenesis.* Ed. P. O. Ts'O and J. A. Di Paolo. New York: Marcell Dekker. p. 61.

MONTESANO, R., BARTSCH, H. & TOMATIS, L. (1976) Screening Tests in Chemical Carcinogenesis. *IARC/WHO Scient. Publ.* 12.

OESCH, F., BENTLEY, P. & GLATT, H. R. (1976) Prevention of Benzo(a)pyrene-induced Mutagenicity by Homogeneous Epoxide Hydratase. *Int. J. Cancer*, **18**, 448.

PURCHASE, I. F. H. & LEFEVRE, P. (1975) Rapid Tests for Carcinogens. *Chemy. Ind.*, **10**, 415.

PURCHASE, I. F. H., LONGSTAFF, E., ASHBY, J. A., ANDERSON, D., LEFEVRE, P. A. & WESTWOOD, F. R. (1976) Evaluation of Six Short Term Tests for Detecting Organic Chemical Carcinogens and Recommendations for Their Use. *Nature*, **264**, 624.

STOLTZ, D. R., POIRIER, L. A., IRVING, C. C., STICH, H. F., WEISBURGER, J. H. & GRICE, H. C. (1974) Evaluation of Short Term Tests for Carcinogenicity. *Toxicol. Appl. Pharmacol.*, **29**, 157.

STYLES, J. A. (1977) A Method for Detecting Carcinogenic Organic Chemicals using Mammalian Cells in Culture. *Br. J. Cancer*, **36**, 558.

WEISBURGER, J. H. & WILLIAMS, G. M. (1975) Metabolism of Chemical Carcinogens. In *Cancer* 1, *Etiology: Chemical and Physical Carcinogenesis* Ed. F. F. Becker. New York: Plenum Press. p. 185.

WHO/IARC PUBLICATIONS (1972–75) Evaluation of Carcinogenic Risk of Chemicals to Man. *IARC Monographs* **1**, 9.

WILLIAMS, D. J. & RABIN, B. R. (1971) Disruption by Carcinogens of the Hormone-dependent Association of Membranes with Polysomes. *Nature*, **232**, 102.

WYNDER, E. L. & MABUCHI, K. (1972) Etiological and Preventative Aspects of Human Cancer. *Preventive Med.*, **1**, 300.